# NSUN3‐Associated m5C Modification of tRF5‐GlyTCC Suppresses Colorectal Cancer Progression via RPL23‐Linked Nucleolar Stress and p53‐Associated Autophagy and Enhances 5‐FU Sensitivity

**DOI:** 10.1002/advs.78345

**Published:** 2026-10-22

**Authors:** Xinhui Lv, Can Tang, Feiyang Song, Yu Wu, Yafang Chen, Sicong Liu, Ziwen Yuan, Guihua Wang, Xudong Wang

**Affiliations:** ^1^ Department of Laboratory Medicine Affiliated Hospital of Nantong University Nantong Jiangsu China; ^2^ Medical School of Nantong University Nantong Jiangsu China

**Keywords:** 5‐FU, autophagy, CRC, m5C, NSUN3, nucleolar stress, RPL23, tsRNA

## Abstract

tRNA‐derived small RNAs (tsRNAs) regulate gene expression and cellular stress responses in cancer, but their roles in colorectal cancer (CRC) progression and chemotherapy response remain incompletely understood. Dysregulated tsRNAs in CRC were identified by RNA sequencing. The functions of tRF5‐GlyTCC were evaluated using in vitro assays and an in vivo xenograft model. Its targets and molecular effects were investigated by bioinformatic analyses, luciferase reporter assays, western blotting, and immunofluorescence. m5C modification was assessed by MeRIP‐qPCR. tRF5‐GlyTCC was downregulated in CRC tissues and associated with advanced disease and poor prognosis. Restoration of tRF5‐GlyTCC suppressed CRC cell growth and migration in vitro and tumor growth in vivo. tRF5‐GlyTCC interacted with the RPL23 3′UTR and was associated with altered ribosomal homeostasis, nucleolar stress, p53 signaling, and autophagy. Restoration of tRF5‐GlyTCC enhanced 5‐FU sensitivity, while its agomir increased the antitumor effect of 5‐FU in vivo. NSUN3‐mediated m5C modification contributed to tRF5‐GlyTCC stability. NSUN3‐mediated m5C modification contributes to tRF5‐GlyTCC stability, which is associated with ribosomal homeostasis, nucleolar stress responses, and p53 signaling. This network contributes to tumor suppression and chemotherapy response in CRC.

## Graphical abstract

This study identifies that NSUN3‐mediated m5C modification stabilizes tRF5‐GlyTCC, linking ribosomal homeostasis, nucleolar stress responses, and p53 signaling changes. This regulatory axis suppresses colorectal cancer and enhances chemotherapy response to 5‐FU, positioning tRF5‐GlyTCC as a potential therapeutic target for improving drug sensitivity in CRC.

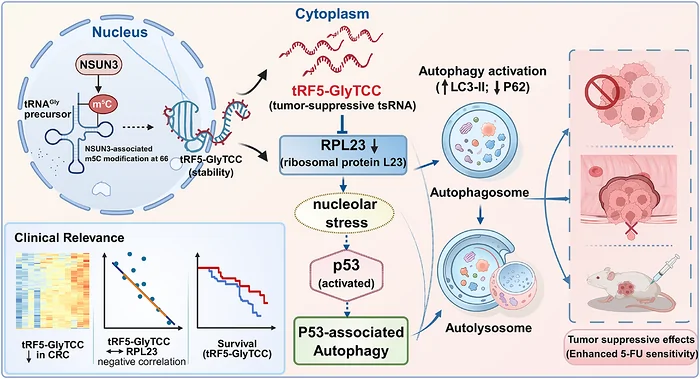

## 1 Introduction

Colorectal cancer (CRC) is one of the most prevalent malignancies worldwide and remains a leading cause of cancer‐related mortality [1, 2]. Tumor heterogeneity, metastatic potential, and variable responses to chemotherapy, particularly 5‐fluorouracil (5‐FU)‐based regimens [3, 4], underscore the need to identify robust biomarkers and better understand the molecular mechanisms underlying CRC progression and therapeutic response.

Small non‐coding RNAs (sncRNAs) have emerged as important regulators of gene expression in cancer, contributing to tumor initiation, progression, and therapeutic response [5, 6, 7]. Among them, transfer RNA‐derived small RNAs (tsRNAs) are a class of regulatory RNAs generated through precise endonucleolytic cleavage of precursor or mature tRNAs rather than random degradation [8, 9]. Based on their cleavage sites, tsRNAs can be broadly classified into 5′‐tRFs, 3′‐tRFs, and internal tRFs (i‐tRFs), which differ in length, sequence composition, and structural features and may therefore exhibit distinct stability and functional properties [10]. tsRNA biogenesis is influenced by RNA modifications, particularly 5‐methylcytosine (m5C), which can affect tRNA stability and susceptibility to cleavage and consequently influence tsRNA production and abundance [11].

Functionally, tsRNAs exert their effects through diverse molecular mechanisms. Some tsRNAs associate with Argonaute proteins and regulate target mRNAs in a miRNA‐like manner, resulting in translational repression or mRNA degradation [12]. Other tsRNAs interact with ribosomal components or translation initiation factors and may influence translation initiation or ribosome assembly [5]. Under cellular stress, certain tsRNAs can interact with translation‐related factors, including eIF4G and eIF4A, and promote stress granule formation, thereby contributing to translational repression [10]. These findings highlight the potential of tsRNAs to integrate RNA regulation with translational and cellular stress responses.

Among these tsRNAs, tRF5‐GlyTCC (tRF5‐23‐GlyTCC‐2) is a 5′‐derived fragment originating from tRNA‐Gly‐TCC. Although tRF5‐GlyTCC has been identified in high‐throughput sequencing datasets, its biological function in CRC remains largely unexplored [13]. Given the involvement of tsRNAs in translational regulation and cellular stress responses, and the close relationship between ribosomal perturbations and nucleolar stress, characterization of tRF5‐GlyTCC may provide further insight into RNA‐mediated regulatory mechanisms in CRC.

RPL23, a component of the 60S ribosomal subunit, has been implicated in the regulation of p53 signaling [14, 15, 16]. RPL23 has been reported to interact with MDM2 and to modulate p53 stability by influencing MDM2‐mediated ubiquitination [14]. More broadly, accumulating evidence indicates that ribosomal proteins can affect p53 signaling in a context‐dependent manner, including through cellular responses associated with perturbations in ribosomal homeostasis and nucleolar stress [15, 17]. These observations raise the possibility that altered expression of ribosomal proteins may link tsRNA‐mediated regulation to stress‐responsive signaling in cancer cells.

p53 is also involved in the regulation of autophagy‐related genes and cellular stress responses and may contribute to cellular adaptation to adverse conditions [18, 19]. In the context of cancer therapy, autophagy has been implicated in the modulation of chemotherapy sensitivity. Stress‐associated autophagic responses may influence the balance between cell survival and cell death and thereby affect the response to chemotherapeutic agents, including 5‐FU [20, 21, 22]. Thus, the interplay among ribosomal homeostasis, nucleolar stress, p53 signaling, and autophagy may represent an important regulatory context for chemotherapy response in CRC.

RNA modifications provide an additional layer of regulation of tsRNA biogenesis and function [11, 23]. Among these modifications, m5C, which is catalyzed by members of the NSUN family of methyltransferases, including NSUN2, NSUN3, and NSUN6, has important roles in tRNA stability and metabolism. m5C modification can affect the susceptibility of tRNAs to endonucleolytic cleavage and thereby influence the production and abundance of tsRNAs [24, 25]. Increasing evidence suggests that dysregulation of m5C‐associated enzymes contributes to tumor progression by altering RNA stability and small RNA biogenesis [26]. Analysis of CRC datasets revealed that NSUN2 and NSUN6 were upregulated, whereas NSUN3 was significantly downregulated. Notably, tRF5‐GlyTCC was also reduced in CRC tissues, showing a similar expression pattern to NSUN3. Based on this data‐driven association and the relatively unexplored role of NSUN3 in tsRNA regulation, we hypothesized that NSUN3 may be involved in the regulation of tRF5‐GlyTCC through an m5C‐dependent mechanism in CRC.

In this study, we systematically investigated the expression pattern, clinical significance, biological function, and regulatory mechanisms of tRF5‐GlyTCC in CRC. Through high‐throughput sequencing and subsequent validation, tRF5‐GlyTCC was identified as a significantly downregulated tsRNA in CRC tissues. Functional assays demonstrated that restoration of tRF5‐GlyTCC suppressed CRC cell proliferation and migration in vitro and reduced tumor growth in vivo. Mechanistically, tRF5‐GlyTCC was associated with reduced RPL23 expression and changes in nucleolar stress‐related p53 signaling and autophagy, accompanied by enhanced sensitivity to 5‐FU. Furthermore, NSUN3‐mediated m5C modification contributed to the regulation of tRF5‐GlyTCC stability, providing a potential epitranscriptomic explanation for the dysregulation of this tsRNA in CRC.

Collectively, our findings identify tRF5‐GlyTCC as a previously uncharacterized tsRNA with tumor‐suppressive activity and potential diagnostic and therapeutic relevance in CRC. This study expands the understanding of tsRNA biology in CRC and identifies a regulatory framework involving NSUN3/m5C, tRF5‐GlyTCC, RPL23, and nucleolar stress‐associated p53 signaling that may contribute to tumor progression and 5‐FU response.

## 2 Materials and Methods

### 2.1 Clinical Samples

From September 2020 to June 2023, 99 paired colorectal cancer (CRC) and adjacent non‐tumor tissue samples were collected from patients who underwent radical surgical resection at the Affiliated Hospital of Nantong University. Patient recruitment, sample collection, and ethical compliance procedures are consistent with our previous research [13]. All patients were pathologically diagnosed with CRC and had not received preoperative chemotherapy or radiotherapy. The study was approved by the Ethics Committee of the Affiliated Hospital of Nantong University (Approval No. 2019‐L053) and was conducted in accordance with the principles of the Declaration of Helsinki. Written informed consent was obtained from all participants before sample collection.

### 2.2 Clinical Follow‐Up

Clinical information and follow‐up data were obtained through medical record review and telephone follow‐up. Overall survival (OS) was defined as the interval from the date of radical surgery to the date of death or the last follow‐up. The median follow‐up period was 36 months (range, 4–36 months), during which 65 deaths were recorded. Clinical and follow‐up information was systematically entered into an electronic database for subsequent statistical and survival analyses.

### 2.3 Correlation Analysis

Pearson correlation analysis was performed to evaluate the relationships between tRF5‐GlyTCC expression and the expression levels of RPL23 or NSUN3 in paired CRC and adjacent non‐tumor tissues. Correlation analyses were performed using the 97 paired tissue samples with complete expression data.

### 2.4 Survival Analysis

The prognostic association of NSUN3 expression was evaluated using the publicly available Kaplan–Meier Plotter platform. Patients were stratified into high‐ and low‐expression groups according to the median NSUN3 expression level. Kaplan–Meier survival curves were generated, and differences between groups were evaluated using the log‐rank test. Hazard ratios (HRs), 95% confidence intervals (CIs), and corresponding P values were obtained from the Kaplan–Meier Plotter platform. Subgroup analyses according to p53 mutation status were also performed using the platform.

### 2.5 Cell Culture

Human CRC cell lines DLD1, SW620, HCT116, SW480, and SW1116, as well as the normal human colonic epithelial cell line NCM460, were obtained from the Cell Bank of the Chinese Academy of Sciences (Shanghai, China). Cells were cultured under standard conditions at 37°C in a humidified atmosphere containing 5% CO_2_ using DMEM or RPMI‐1640 medium supplemented with 10% fetal bovine serum (FBS) and 1% penicillin‐streptomycin, as appropriate. Cell lines were authenticated by short tandem repeat (STR) profiling, with the most recent authentication performed in September 2021, and were used between passages 5 and 20.

### 2.6 RNA Extraction and Quantitative Real‐Time PCR

Total RNA was extracted using TRIzol reagent according to the manufacturer's instructions. For mRNA and precursor tRNA analyses, 1 µg of total RNA was reverse‐transcribed into cDNA. Quantitative real‐time PCR (RT‐qPCR) was performed using ChamQ Universal SYBR qPCR Master Mix on an Applied Biosystems 7500 Real‐Time PCR System. Each 20 µL reaction contained 10 µL of SYBR qPCR Master Mix, 1 µL of each primer, 5 µL of appropriately diluted cDNA, and 3 µL of nuclease‐free water. tRF5‐GlyTCC and U6 (RNU6B) primers were obtained from RiboBio (Guangzhou, China). U6 was used as the reference gene for tRF5‐GlyTCC quantification. Relative expression levels were calculated using the 2^−^ΔΔCt method. Primer sequences are listed in Table S1.

For RNA extracted from paraffin‐embedded tissue samples, total RNA was isolated using the RNAeasy Tissue RNA Extraction Kit (Beyotime, China). RNA concentration and quality were assessed before reverse transcription and RT‐qPCR analysis. U6 was used as the reference gene.

### 2.7 Precursor tRNA‐Gly‐TCC Quantification

To determine whether NSUN3 depletion affected the abundance of the precursor tRNA‐Gly‐TCC from which tRF5‐GlyTCC is derived, precursor tRNA‐Gly‐TCC levels were measured by RT‐qPCR. Total RNA was extracted from CRC cells following the indicated transfections and subjected to reverse transcription. The precursor tRNA‐Gly‐TCC was quantified using a primer pair specifically designed for the precursor sequence and listed in Table S1. The primer design targeted the precursor tRNA sequence rather than the mature 23‐nucleotide tRF5‐GlyTCC sequence, thereby allowing assessment of precursor abundance independently of the mature tRF.

### 2.8 Nuclear and Cytoplasmic RNA Fractionation

Nuclear and cytoplasmic fractions were isolated using a Nuclear and Cytoplasmic Protein Extraction Kit (Beyotime, China) according to the manufacturer's instructions with minor modifications. Briefly, cells were lysed in cytoplasmic extraction buffer, and the cytoplasmic supernatant was separated from the nuclear pellet by centrifugation. The nuclear pellet was subsequently lysed to obtain the nuclear fraction.

Importantly, the fractionation buffers were used only for subcellular separation. RNA was subsequently extracted separately from the cytoplasmic and nuclear fractions using TRIzol reagent (Invitrogen). TRIzol contains strong denaturing agents that rapidly inactivate endogenous RNases and thereby help preserve RNA integrity. This workflow is consistent with previously reported approaches [27], in which RNA was extracted from fractionated subcellular compartments using TRIzol reagent.

All procedures were performed on ice, and each fraction was immediately mixed with TRIzol reagent following separation to minimize potential RNA degradation.

The efficiency of nuclear–cytoplasmic fractionation was verified by RT‐qPCR analysis of the nuclear marker U6 and the cytoplasmic marker GAPDH mRNA, which showed the expected enrichment patterns.

### 2.9 Cell Transfection

For transient transfection, cells were seeded in 6‐well plates and transfected at approximately 60–70% confluence using Lipofectamine 3000 reagent according to the manufacturer's instructions. The tRF5‐GlyTCC mimic and inhibitor were purchased from RiboBio (Guangzhou, China), while the corresponding negative‐control mimic and inhibitor were obtained from GenePharma (Shanghai, China). Cells were collected or subjected to subsequent functional assays at the indicated time points, generally 24–48 h after transfection.

### 2.10 Colony Formation Assay

Transfected cells were dissociated using trypsin, counted, and seeded into 6‐well plates at a density of 1 × 10^3^ cells per well. Cells were cultured for approximately 14 days, with the culture medium replaced every 4 days. Colonies were fixed with 4% paraformaldehyde (BioSharp, China) for 15 min and stained with 0.1% crystal violet for 20 min. After washing with PBS and air‐drying, colonies containing more than 50 cells were counted.

### 2.11 CCK‐8 Assay and Drug Sensitivity Analysis

Cell viability was evaluated using a Cell Counting Kit‐8 (CCK‐8) assay (BioSharp, China). 48 h after transfection, cells were seeded into 96‐well plates at a density of 3 × 10^3^ cells per well. CCK‐8 reagent was added and incubated for 2 h at 37°C, and absorbance was measured at 450 nm. For proliferation assays, measurements were performed at 24 h intervals in triplicate.

For 5‐fluorouracil (5‐FU) dose‐response experiments, cells were treated with increasing concentrations of 5‐FU, and cell viability was determined using the CCK‐8 assay. For drug‐response analyses presented in Figure 8, the viability data were log_2_‐transformed before statistical analysis. Half‐maximal inhibitory concentrations (IC_50_) were calculated by nonlinear regression using GraphPad Prism.

For experiments evaluating the contribution of autophagy to 5‐FU sensitivity, chloroquine (CQ; HY‐17589) was used at a final concentration of 10 µm and added 2 h before 5‐FU treatment. Cell viability was subsequently evaluated using the CCK‐8 assay under the indicated treatment conditions.

### 2.12 EdU Incorporation Assay

Cell proliferation was evaluated using an EdU Cell Proliferation Assay Kit (Beyotime, China). Transfected cells were seeded in 12‐well plates and incubated with 300 µL of EdU working solution for 2 h. Cells were then fixed with 4% paraformaldehyde for 15 min and permeabilized with 0.3% Triton X‐100 for 15 min. Nuclei were counterstained with Hoechst 33258 (1 µg/mL) for 15 min. Fluorescence images were acquired using a fluorescence microscope, and EdU‐positive cells were quantified.

### 2.13 Transwell Migration Assay

Cell migration was assessed using Transwell chambers. Briefly, 600 µL of medium containing 20% FBS was added to the lower chamber, while 8 × 10^4^ transfected cells suspended in serum‐free medium were seeded into the upper chamber. After 48 h of incubation, cells that had migrated to the lower surface of the membrane were fixed and stained with crystal violet. Images were captured using a light microscope, and migrated cells were quantified.

### 2.14 RNA Sequencing and Identification of Differentially Expressed tsRNAs

For small RNA sequencing, six paired CRC and adjacent non‐tumor tissues were analyzed by Aksomics (Shanghai, China). For transcriptome sequencing, CRC cells transfected with the tRF5‐GlyTCC mimic or corresponding negative control were directly lysed in TRIzol reagent and stored at low temperature before sequencing by Novogene (Beijing, China).

Differentially expressed tsRNAs were initially identified using a P value <0.05 and a fold change (FC) <0.5 for downregulated tsRNAs. Because of the limited sample size of the discovery cohort, the P value was used as the primary statistical criterion, while false‐discovery rate (FDR) or q values were also provided in Table S2. Candidate tsRNAs were ranked mainly according to fold change, and the three most prominent candidates were selected for subsequent validation. Detailed differential expression results are provided in Table S2.

### 2.15 Dual‐Luciferase Reporter Assay

The wild‐type (WT) and mutant (MUT) sequences containing the predicted tRF5‐GlyTCC‐binding site within the 3′ untranslated region (3′UTR) of RPL23 were cloned into the pmirGLO dual‐luciferase reporter vector. CRC cells were co‐transfected with the reporter constructs and tRF5‐GlyTCC mimic or the corresponding negative control. After 48 h, firefly and Renilla luciferase activities were measured using the Dual‐Luciferase Reporter Assay System (Promega, USA) according to the manufacturer's instructions. Relative luciferase activity was calculated by normalizing firefly luciferase activity to Renilla luciferase activity.

### 2.16 Western Blotting

Cells or tissues were lysed in RIPA buffer supplemented with PMSF (SolarBio, China). Protein concentrations were determined using a bicinchoninic acid (BCA) assay. Equal amounts of protein were separated by SDS‐PAGE and transferred onto 0.45 µm PVDF membranes (Millipore, USA). Membranes were blocked for 2 h at room temperature and incubated with primary antibodies overnight at 4°C, followed by incubation with the corresponding secondary antibodies for 1.5 h. Protein signals were visualized and quantified using ImageJ software.

Antibodies against GAPDH, RPL23, and p53 were obtained from Proteintech (China). Antibodies against LAMP1, LAMP2, LC3A/II, p62, ATG5, and NSUN3 were obtained from UpingBio (China).

### 2.17 Immunohistochemistry

Tumor tissues were fixed in 4% paraformaldehyde, embedded in paraffin, and sectioned. Sections were deparaffinized in xylene and rehydrated through a graded ethanol series. Antigen retrieval was performed in citrate buffer (pH 6.0) using microwave heating at high power for 2.5 min, followed by low power for 15 min. The sections were then allowed to cool naturally to room temperature. Endogenous peroxidase activity was blocked with 3% hydrogen peroxide for 20 min in the dark, followed by blocking of nonspecific binding with 5% BSA for 20 min at room temperature.

Sections were incubated overnight at 4°C with primary antibodies against Ki‐67 (1:200), PCNA (1:200), RPL23 (1:200), and p53 (1:500). After washing with PBS, sections were incubated with HRP‐conjugated secondary detection reagents, including the enhancer and secondary antibody, for 30 min each at room temperature. Immunoreactivity was visualized using DAB substrate, followed by counterstaining with hematoxylin. Sections were subsequently dehydrated, cleared in xylene, and mounted. Images were captured using an optical microscope at 20× magnification.

For quality control, negative controls were processed without the primary antibody, and appropriate positive control tissues were included to verify staining specificity.

### 2.18 Transmission Electron Microscopy

For transmission electron microscopy (TEM), transfected cells were harvested by trypsinization and collected by centrifugation. Cell pellets were fixed in 1 mL of electron microscopy fixative at 4°C for 2 h. Prewarmed 1% agarose was then added to the pellets, which were allowed to solidify. The resulting samples were retrieved and maintained at 4°C until further processing for TEM analysis.

### 2.19 Xenograft Tumor Model

All animal experiments were approved by the Institutional Animal Care and Use Committee of Nantong University (Approval No. S20250821‐001). Sixteen 4‐week‐old female BALB/c nude mice were obtained from the Experimental Animal Center of Nantong Medical College and acclimatized for 1 week under specific pathogen‐free conditions.

HCT116 cells (2.5 × 10^6^ cells in 100 µL PBS) were subcutaneously injected into the right axilla of each mouse. When tumors were established, mice were randomly divided into four groups (n = 4 per group): Group 1, agomir NC + PBS; Group 2, tRF5‐GlyTCC agomir + PBS; Group 3, agomir NC + 5‐FU; and Group 4, tRF5‐GlyTCC agomir + 5‐FU. The tRF5‐GlyTCC agomir and corresponding NC agomir were purchased from GenePharma (Shanghai, China). Starting on day 9 after tumor cell inoculation, 20 µg of agomir or NC agomir was administered by intratumoral injection every 3 days. Starting on day 15, 5‐FU was administered intraperitoneally at 25 mg/kg every 2 days for a total of four administrations. An equivalent volume of PBS (200 µL) was administered to the corresponding control groups.

Tumor length and width were measured every 3 days, and tumor volume was calculated using the formula: volume = length × width^2^/2. At the end of the experiment, mice were euthanized by CO_2_ asphyxiation, and tumors were excised, weighed, and either fixed in 4% paraformaldehyde or stored at low temperature for subsequent molecular analyses.

### 2.20 m5C RNA Methylation Immunoprecipitation and RT‐qPCR

m5C RNA methylation was analyzed using the BersinBio m5C Methylated RNA Immunoprecipitation (m5C MeRIP) Kit according to the manufacturer's instructions. Approximately 2 × 10^7^ cells were collected, and total RNA was extracted using TRIzol reagent. RNA was fragmented to an average length of approximately 100 nt by incubation at 94°C and subsequently resuspended in immunoprecipitation buffer supplemented with RNase inhibitors.

An aliquot of fragmented RNA was retained as input, while the remaining RNA was divided for immunoprecipitation using the kit‐provided m5C antibody or the corresponding normal IgG control. Immunoprecipitation was performed at 4°C for 4 h. Pre‐blocked Protein A/G magnetic beads were subsequently added and incubated for 1 h. The beads were washed, and the immunoprecipitated RNA was eluted and purified by phenol‐chloroform extraction followed by ethanol precipitation. Equal volumes of recovered RNA were subjected to reverse transcription and RT‐qPCR using SYBR Green chemistry.

### 2.21 Immunofluorescence Analysis of Nucleolar Stress

For immunofluorescence analysis, cells were seeded on coverslips and transfected with the indicated oligonucleotides for 48 h. For the genetic interaction experiments, cells were divided into four groups: NC, tRF5‐GlyTCC inhibitor, siRPL23, and siRPL23 + tRF5‐GlyTCC inhibitor.

Cells were fixed with 4% paraformaldehyde for 15 min and permeabilized with 0.1% Triton X‐100 for 10 min. After blocking with 5% BSA for 1 h, cells were incubated with an anti‐NPM1 antibody (Abcam, UK) overnight at 4°C, followed by incubation with an Alexa Fluor‐conjugated secondary antibody for 1 h. Nuclei were counterstained with DAPI (Beyotime, China). Images were acquired using a confocal fluorescence microscope. Nucleolar stress was assessed based on the redistribution of NPM1 from the nucleolus to the nucleoplasm.

### 2.22 p53‐GFP Reporter Assay

To further evaluate changes in p53 activity, cells were co‐transfected with a p53‐GFP reporter plasmid (Yeasen, China) and tRF5‐GlyTCC mimic or the corresponding negative control using Lipofectamine 3000. After 48 h, GFP fluorescence was examined using a fluorescence microscope. GFP fluorescence intensity was quantified using ImageJ software.

### 2.23 SUnSET Assay

Global protein synthesis was assessed using the surface sensing of translation (SUnSET) assay. 48 h after transfection, cells were incubated with puromycin (5 µg/mL; MCE, China) for 30 min at 37°C. Cells were then fixed with 4% paraformaldehyde for 15 min and permeabilized with 0.1% Triton X‐100 for 10 min. After blocking with 5% BSA for 1 h, cells were incubated with an anti‐puromycin antibody overnight at 4°C, followed by incubation with a fluorescent secondary antibody for 1 h. Nuclei were counterstained with DAPI, and fluorescence images were acquired using a confocal microscope. Puromycin‐associated fluorescence intensity was quantified using ImageJ software as an indicator of global protein synthesis.

### 2.24 Autophagic Flux Assessment by Chloroquine Treatment

To assess whether the observed changes in autophagy‐related proteins were associated with altered autophagic flux, chloroquine (CQ; HY‐17589) was used to inhibit lysosomal degradation. Cells were treated with 10 µM CQ for 2 h before subsequent analysis. Following the indicated treatments, cells were lysed and subjected to Western blotting for LC3A/II and p62. The changes in LC3‐II and p62 levels in the presence or absence of CQ were evaluated to assess autophagic flux.

### 2.25 RNA Stability Assay

To assess the effect of NSUN3 depletion on tRF5‐GlyTCC stability, DLD1 and HCT116 cells were transfected with siNC or siNSUN3 for 48 h and subsequently treated with actinomycin D (ActD; 5 µg/mL) to inhibit transcription. Cells were harvested at 0, 2, 4, and 8 h after ActD treatment. Total RNA was extracted, and tRF5‐GlyTCC levels were determined by RT‐qPCR. Expression levels were normalized to the corresponding 0 h value. RNA decay curves were generated by nonlinear regression, and the half‐life of tRF5‐GlyTCC was calculated using GraphPad Prism.

### 2.26 Statistical Analysis

All quantitative data are presented as mean ± standard deviation (SD) from at least three independent experiments unless otherwise indicated. Statistical analyses were performed using SPSS version 20.0 and GraphPad Prism version 10.1.2. Comparisons between two groups were performed using a two‐tailed Student's t‐test. Comparisons among multiple groups were performed using one‐way analysis of variance (ANOVA) followed by an appropriate post hoc multiple‐comparisons test. For experiments involving two independent factors, two‐way ANOVA followed by the indicated multiple‐comparisons test was used. Categorical variables were analyzed using the chi‐square test. Survival distributions were compared using the Kaplan–Meier method and log‐rank test. For the 5‐FU dose‐response experiments involving CQ, two‐way ANOVA followed by Šídák's multiple‐comparisons test was used where appropriate. All statistical tests were two‐sided, and P < 0.05 was considered statistically significant.

## 3 Results

### 3.1 tRF5‐GlyTCC is Significantly Downregulated in CRC Tissues and is Associated with Poor Clinical Outcome

To comprehensively characterize the involvement of transfer RNA‐derived small RNAs (tsRNAs) in colorectal cancer (CRC), we first performed high‐throughput sequencing of six paired CRC and adjacent non‐tumor tissues. As shown in Figure 1A–C, global expression profiling identified a subset of tsRNAs that were differentially expressed between CRC and matched adjacent tissues. Using a significance threshold of < 0.05 and a fold change (FC) < 0.5, significantly downregulated tsRNAs were identified. These candidates were subsequently ranked in ascending order according to their FC values (Table S2).

**FIGURE 1:**
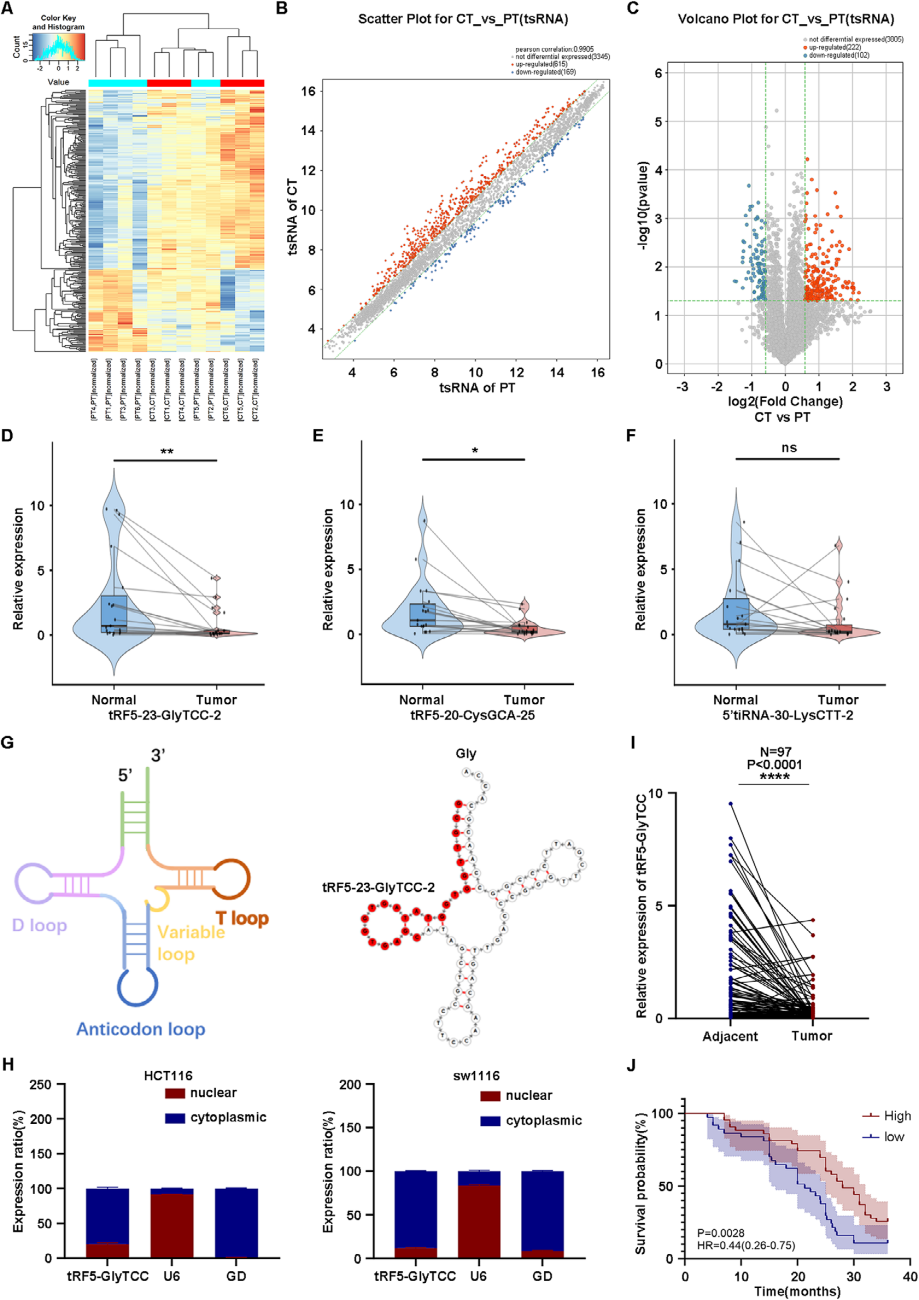
Identification and clinical relevance of tRF5‐GlyTCC in colorectal cancer. (A)Heatmap of differentially expressed tsRNAs between colorectal cancer (CRC) tissues and matched adjacent non‐tumor tissues. (B) Scatter plot showing the distribution of tsRNA expression between CRC and matched adjacent non‐tumor tissues. (C) Volcano plot of tsRNA expression profiles. Red and blue dots indicate significantly upregulated and downregulated tsRNAs, respectively (*P* < 0.05). (D–F) RT‐qPCR validation of tRF5‐23‐GlyTCC‐2 (D), tRF5‐20‐CysGCA‐25 (E), and 5′‐tsRNA‐30‐LysCTT‐2 (F) in 20 paired CRC and matched adjacent non‐tumor tissues. Data are presented as paired violin plots with individual data points; paired samples are connected by lines. Statistical significance was determined using a two‐tailed paired Student's *t*‐test. (G) Schematic illustration of the genomic origin of tRF5‐GlyTCC derived from the 5′ end of tRNA‐Gly‐TCC. (H) Subcellular localization of tRF5‐GlyTCC determined by nuclear–cytoplasmic fractionation. Fractionation efficiency was evaluated by RT‐qPCR detection of the nuclear marker U6 and the cytoplasmic marker GAPDH mRNA. (I) Expression levels of tRF5‐GlyTCC in CRC tissues and matched adjacent non‐tumor tissues from an expanded cohort (*n* = 97). (J) Kaplan–Meier analysis of overall survival according to tRF5‐GlyTCC expression levels. *P* < 0.05, ^*^ *P* < 0.01, ^**^ *P* < 0.001, ^***^ *P* < 0.0001.

The three most prominently downregulated tsRNAs, tRF5‐23‐GlyTCC‐2 (hereafter referred to as tRF5‐GlyTCC), tRF5‐20‐CysGCA‐25, and 5′‐tsRNA‐30‐LysCTT‐2, were selected for further validation. RT‐qPCR analysis confirmed that all three tsRNAs were significantly downregulated in CRC tissues compared with matched adjacent non‐tumor tissues (Figure 1D–F), consistent with the sequencing results. Among these candidates, tRF5‐GlyTCC exhibited the most pronounced reduction and was therefore selected for subsequent functional and mechanistic investigations.

tRF5‐GlyTCC is derived from the 5′ terminal region of tRNA‐Gly‐TCC (Figure 1G), and its mature sequence was confirmed by Sanger sequencing (Figure S1A). Subcellular fractionation followed by RT‐qPCR showed that tRF5‐GlyTCC was predominantly localized in the cytoplasmic fraction (Figure 1H), consistent with a potential post‐transcriptional regulatory function.

To further evaluate its clinical relevance, we expanded the analysis to a larger cohort of 97 paired CRC and adjacent non‐tumor tissues. Consistent with the sequencing and initial validation results, tRF5‐GlyTCC expression was significantly lower in CRC tissues than in matched adjacent tissues (Figure 1I). Similarly, tRF5‐GlyTCC expression was significantly lower in CRC cell lines than in the normal colonic epithelial cell line NCM460 (Figure S1B).

Survival analysis showed that patients with low tRF5‐GlyTCC expression had significantly shorter overall survival than those with high expression (Figure 1J). Clinically, reduced tRF5‐GlyTCC expression was associated with more aggressive tumor characteristics, including higher T stage, lymph node metastasis, and advanced TNM stage (Table 1).

**TABLE 1:** Clinicopathological analysis of tRF5‐GlyTCC in CRC patients (n = 97).

Parameter		No. of patients	tRF5‐GlyTCC High (n = 49) Low (n = 48)		p Value
Sex	Male	62	28	34	0.160
	Female	35	21	14	
Age (year)	>60	72	37	35	0.770
	≤60	25	12	13	
Tumor size	>5	24	11	13	0.597
	≤5	73	38	35	
Differentiation grade	Well‐moderate	86	47	39	0.050
	Poor	11	2	9	
T stage	T1‐T2	27	19	8	0.015^*^
	T3‐T4	70	30	40	
Lymph node status	Positive	50	20	30	0.033^*^
	Negative	47	29	18	
N stage	N0‐1	90	45	45	1.000
	N2	7	4	3	
TNM stage	I‐II	46	31	15	0.002^**^
	III‐IV	51	18	33	
vascular‐invasion	Positive	34	17	17	0.941
	Negative	63	32	31	
Nerve‐invasion	Positive	25	11	14	0.449
	Negative	72	38	34	

*Note*: Clinical correlations of tRF5‐GlyTCC were analyzed in the same cohort of 97 patients used for RPL23 analysis. TNM: tumor, node metastasis. ^*^ *p* < 0.05; ^**^ *p* < 0.01; ^***^ *p* < 0.001; ^****^ *p* < 0.0001.

Collectively, these findings demonstrate that tRF5‐GlyTCC is markedly downregulated in CRC and that its reduced expression is associated with aggressive clinicopathological features and poor clinical outcome.

### 3.2 tRF5‐GlyTCC Suppresses CRC Cell Proliferation and Migration In Vitro

Given the consistent downregulation of tRF5‐GlyTCC in CRC tissues and cell lines, we next investigated its biological functions. Synthetic tRF5‐GlyTCC mimics and inhibitors were used to modulate endogenous tRF5‐GlyTCC levels, with transfection efficiency confirmed by RT‐qPCR (Figure S2A,B). CCK‐8 assays showed that tRF5‐GlyTCC overexpression significantly reduced cell viability in DLD1 and SW1116 cells, whereas inhibition of tRF5‐GlyTCC increased cell proliferation (Figure 2A).

**FIGURE 2:**
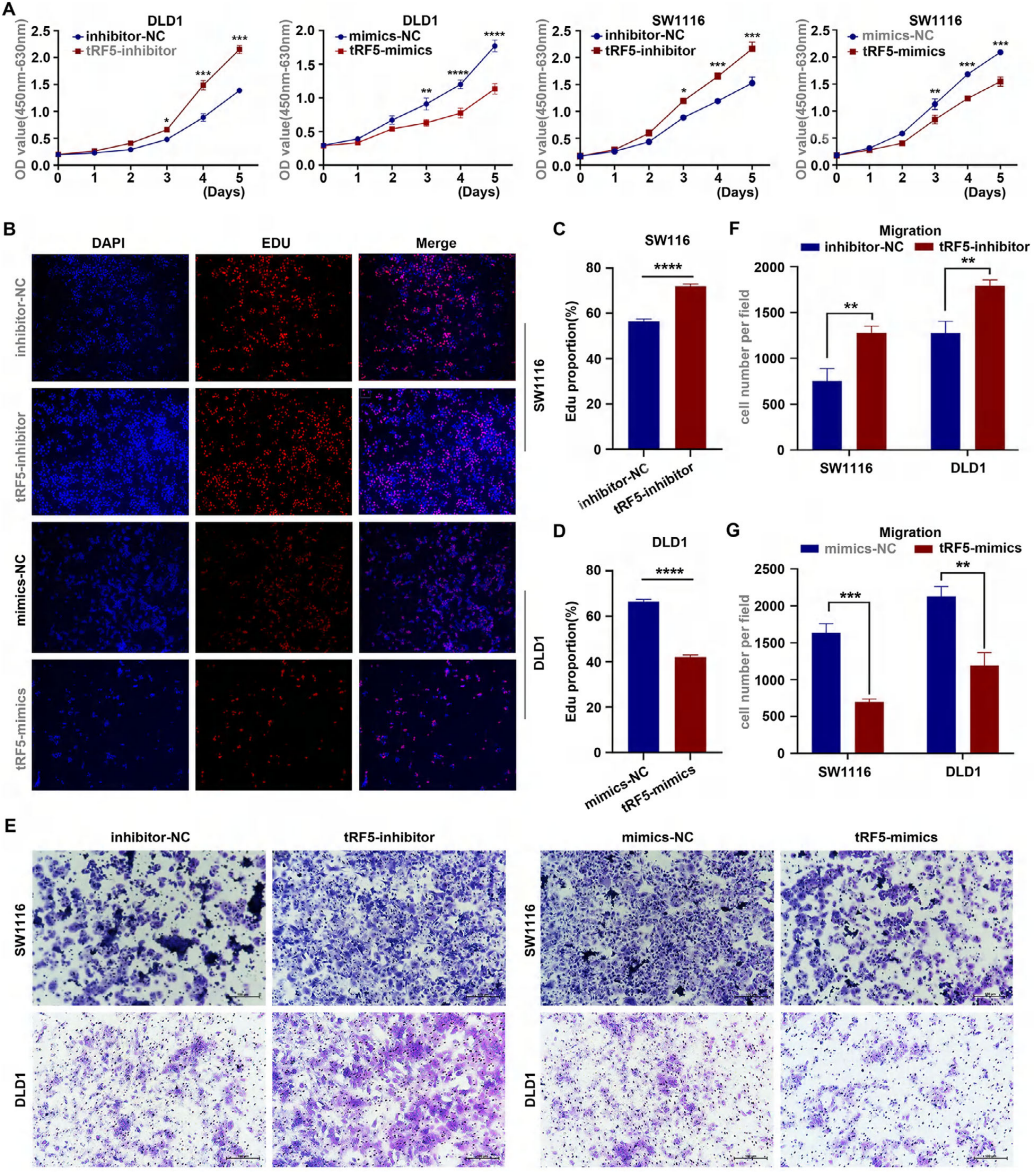
Effects of tRF5‐GlyTCC on colorectal cancer cell proliferation and migration in vitro. (A)Cell viability of DLD1 and SW1116 cells assessed by CCK‐8 assays following transfection with tRF5‐GlyTCC mimic or inhibitor. (B–D) EdU incorporation assays assessing DNA synthesis following gain‐ or loss‐of‐function of tRF5‐GlyTCC. (E–G) Transwell migration assays following modulation of tRF5‐GlyTCC expression. *P* < 0.05, ^*^ *P* < 0.01, ^**^ *P* < 0.001, ^***^ *P* < 0.0001.

EdU incorporation assays further supported these findings. Overexpression of tRF5‐GlyTCC significantly reduced DNA synthesis, whereas inhibition of tRF5‐GlyTCC increased the proportion of proliferating cells (Figure 2B–D). In addition to its effects on cell proliferation, tRF5‐GlyTCC also affected CRC cell migration. Transwell assays showed that tRF5‐GlyTCC overexpression significantly reduced cell migration, whereas inhibition of tRF5‐GlyTCC enhanced migratory capacity (Figure 2E–G).

Collectively, these results support a tumor‐suppressive role for tRF5‐GlyTCC in vitro by restricting CRC cell proliferation and migration.

### 3.3 tRF5‐GlyTCC Directly Targets the 3′UTR of RPL23 and Inhibits its Expression

To investigate the molecular mechanisms underlying the tumor‐suppressive effects of tRF5‐GlyTCC, we sought to identify its downstream targets. Candidate targets were screened by integrating TargetScan and miRanda prediction results with transcriptome profiling of tRF5‐GlyTCC‐overexpressing cells (Figure 3A,B). Three genes, RPL23, NACC1, and DDX5, were consistently identified across the datasets (Figure 3C). RT‐qPCR analysis showed that tRF5‐GlyTCC overexpression most prominently reduced the expression of RPL23 and NACC1 (Figure 3D), suggesting that these genes may be involved in the effects of tRF5‐GlyTCC.

**FIGURE 3:**
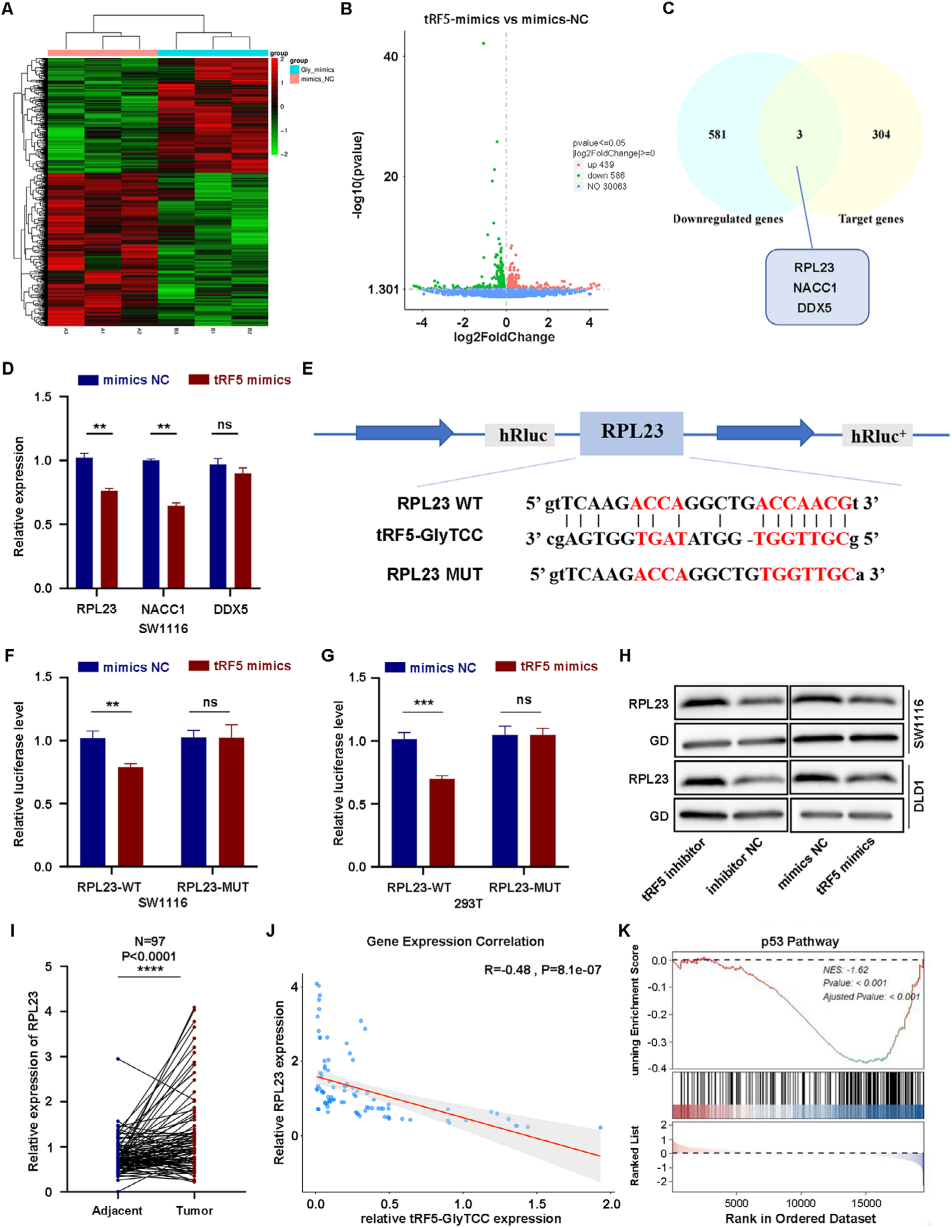
Identification of RPL23 as a downstream target of tRF5‐GlyTCC. (A–B) Schematic overview of the transcriptomic analysis performed in tRF5‐GlyTCC‐overexpressing cells. (C) Venn diagram showing the overlap of candidate target genes identified by integrated bioinformatic analyses. (D) RT‐qPCR analysis of candidate genes following tRF5‐GlyTCC overexpression. (E) Schematic representation of the wild‐type (WT) and mutant (MUT) RPL23 3′UTR luciferase reporter constructs. (F–G) Dual‐luciferase reporter assays in SW1116 and HEK‐293T cells co‐transfected with tRF5‐GlyTCC mimic and WT or MUT RPL23 3′UTR reporters. (H) Western blotting analysis of RPL23 protein levels following tRF5‐GlyTCC overexpression or inhibition. (I) RPL23 expression levels in CRC tissues and matched adjacent non‐tumor tissues. (J) Pearson correlation analysis of tRF5‐GlyTCC and RPL23 expression in 97 paired CRC tissue samples. (K) Pathway enrichment analysis associated with RPL23 expression. *P* < 0.05, ^*^ *P* < 0.01, ^**^ *P* < 0.001, ^***^ *P* < 0.0001.

To determine whether tRF5‐GlyTCC directly interacts with the RPL23 3′UTR, we constructed luciferase reporter vectors containing either the wild‐type (WT) or mutant (MUT) sequence encompassing the predicted tRF5‐GlyTCC‐binding region (Figure 3E). Dual‐luciferase reporter assays showed that tRF5‐GlyTCC mimic significantly reduced the luciferase activity of the RPL23‐WT reporter in both SW1116 and HEK‐293T cells, whereas mutation of the predicted binding region abolished this effect (Figure 3F,G). In contrast, tRF5‐GlyTCC overexpression did not significantly affect the luciferase activity of the NACC1‐WT reporter (Figure S3A,B), supporting the specificity of the interaction with RPL23.

At the protein level, Western blotting showed that tRF5‐GlyTCC overexpression reduced RPL23 protein abundance, whereas inhibition of tRF5‐GlyTCC increased RPL23 expression (Figure 3H), further supporting a direct regulatory relationship between tRF5‐GlyTCC and RPL23.

Clinically, RPL23 expression was significantly higher in CRC tissues than in adjacent non‐tumor tissues (Figure 3I; Figure S3C) and was inversely correlated with tRF5‐GlyTCC expression across the 97 paired tissue samples (Figure 3J). Higher RPL23 expression was associated with advanced T stage, lymph node metastasis, higher TNM stage, and poor differentiation (Table 2), supporting its potential association with malignant CRC phenotypes.

**TABLE 2:** Clinicopathological analysis of RPL23 in CRC patients (n = 97).

Parameter		No. of patients	RPL23 High (n = 49) Low (n = 48)		p Value
Sex	Male	62	30	32	0.577
	Female	35	19	16	
Age (year)	>60	72	35	37	0.524
	≤60	25	14	11	
Tumor size	>5	24	13	11	0.68
	≤5	73	36	37	
Differentiation grade	Well‐moderate	86	39	47	0.012^*^
	Poor	11	10	1	
T stage	T1‐T2	27	9	18	0.036^*^
	T3‐T4	70	40	30	
Lymph node status	Positive	50	31	19	0.02^*^
	Negative	47	18	29	
N stage	N0‐1	90	44	46	0.449
	N2	7	5	2	
TNM stage	I‐II	46	18	28	0.033^*^
	III‐IV	51	31	20	
vascular‐invasion	Positive	34	15	19	0.355
	Negative	63	34	29	
Nerve‐invasion	Positive	25	10	15	0.222
	Negative	72	39	33	

*Note*: Clinical correlations of RPL23 were analyzed in the same cohort of 97 patients used for tRF5‐GlyTCC analysis. TNM, tumor node metastasis; p < 0.05; ^*^ *p* < 0.01; ^**^ *p* < 0.001; ^**^ *p* < 0.0001.

Bioinformatic analysis further indicated that RPL23 expression was associated with p53‐related signaling pathways (Figure 3K; Figure S3D), suggesting that the tRF5‐GlyTCC–RPL23 regulatory relationship may be linked to changes in p53‐associated signaling.

### 3.4 tRF5‐GlyTCC Promotes Autophagy in Association with RPL23‐Associated p53 Signaling

Given the regulatory relationship between tRF5‐GlyTCC and RPL23, we next investigated downstream cellular processes associated with this regulatory relationship. Gene set enrichment analysis (GSEA) of transcriptome data from tRF5‐GlyTCC‐overexpressing cells showed significant enrichment of p53 signaling and autophagy‐related pathways (Figure 4A,B).

**FIGURE 4:**
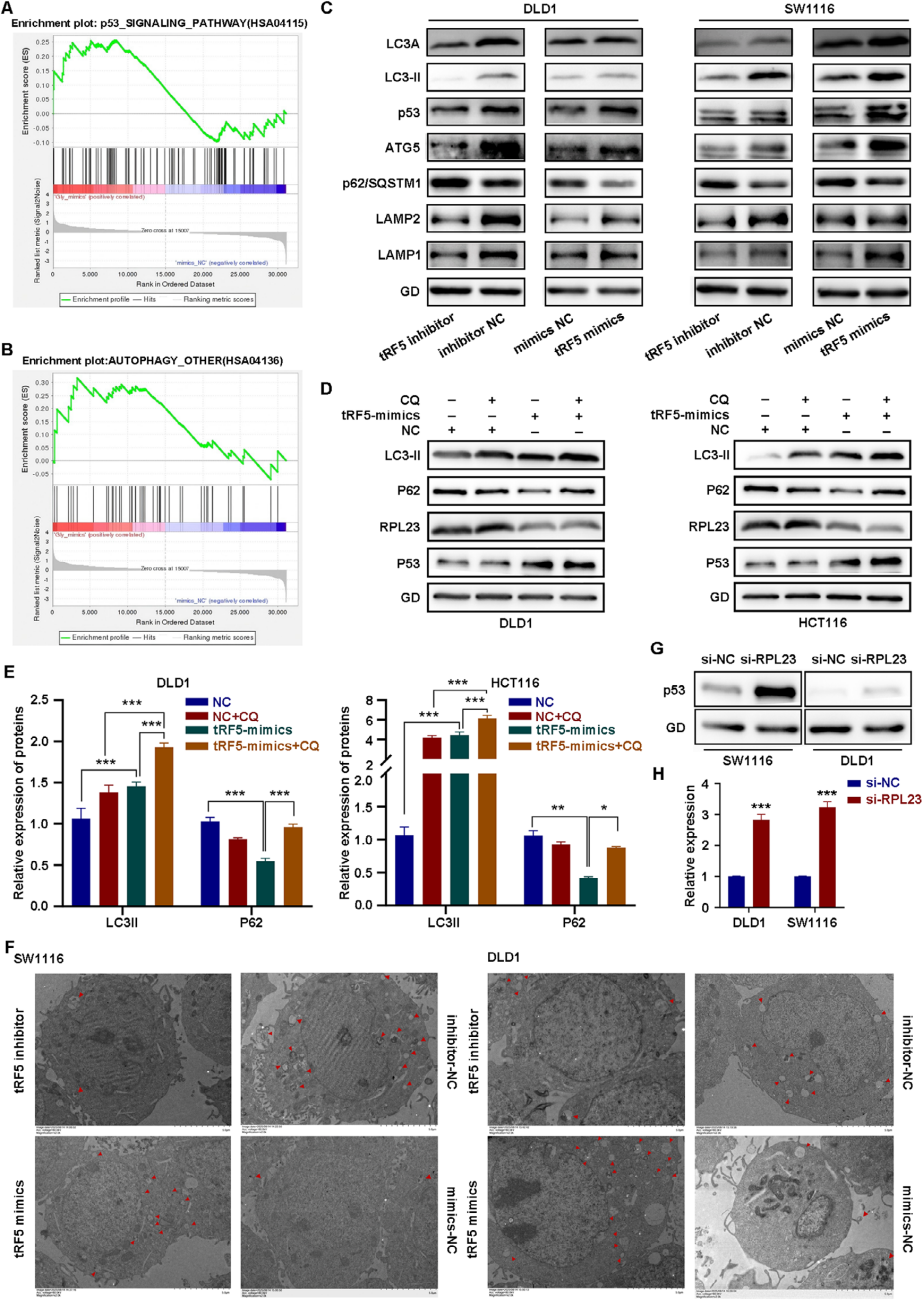
Regulation of p53 signaling and autophagy‐associated responses by tRF5‐GlyTCC. (A–B) Gene set enrichment analysis (GSEA) showing enrichment of p53 signaling and autophagy‐related pathways in tRF5‐GlyTCC‐overexpressing cells. (C) Western blotting analysis of p53 and autophagy‐associated proteins following modulation of tRF5‐GlyTCC expression. (D) Representative Western blotting analysis of LC3‐II, p62, RPL23, and p53 in DLD1 and HCT116 cells transfected with a negative control (NC) mimic or tRF5‐GlyTCC mimic (tRF5‐M), with or without chloroquine (CQ) treatment. (E) Quantification of LC3‐II and p62 protein levels normalized to GAPDH. Data are presented as mean ± SD from independent experiments. Statistical significance was determined using one‐way ANOVA followed by Šídák's multiple‐comparisons test. (F) Representative transmission electron microscopy images showing autophagosome formation in CRC cells following gain‐ or loss‐of‐function of tRF5‐GlyTCC. (G–H) Western blotting analysis of p53 protein levels following RPL23 silencing. *P* < 0.05, ^*^ *P* < 0.01, ^**^ *P* < 0.001.

Western blot analysis showed that tRF5‐GlyTCC overexpression increased p53 protein levels and was accompanied by increased LC3‐II, ATG5, and LAMP1/2 levels and decreased p62 levels. Conversely, inhibition of tRF5‐GlyTCC reduced LC3‐II levels and increased p62 levels (Figure 4C; Figure S4A,B), consistent with changes in autophagy‐associated activity.

To further determine whether the increase in LC3‐II was associated with enhanced autophagic flux rather than impaired lysosomal degradation, we performed chloroquine (CQ) treatment experiments. In both DLD1 and HCT116 cells, CQ treatment further increased LC3‐II levels in tRF5‐GlyTCC‐overexpressing cells compared with tRF5‐GlyTCC overexpression alone. In parallel, the reduction in p62 levels associated with tRF5‐GlyTCC overexpression was attenuated following CQ treatment (Figure 4D,E). These findings support increased autophagic flux following tRF5‐GlyTCC overexpression rather than LC3‐II accumulation attributable solely to impaired lysosomal degradation.

Transmission electron microscopy further provided morphological evidence of increased autophagosome formation following tRF5‐GlyTCC overexpression, whereas autophagosome formation was reduced following tRF5‐GlyTCC inhibition (Figure 4F). In addition, RPL23 silencing was accompanied by increased p53 protein abundance (Figure 4G,H). Together, these findings suggest that RPL23 is associated with p53 signaling and that tRF5‐GlyTCC may influence this signaling context through regulation of RPL23.

Overall, these results support a role for tRF5‐GlyTCC in promoting autophagy in association with RPL23‐related p53 signaling. The molecular basis of the relationship between RPL23, nucleolar stress, and p53 signaling was subsequently investigated.

### 3.5 Further Mechanistic Insights into RPL23‐Associated Nucleolar Stress and p53 Signaling

To further investigate the relationship between RPL23, nucleolar stress, and p53 signaling, we performed genetic interaction experiments focusing on nucleolar stress, global protein synthesis, and p53 activation.

We first examined whether RPL23 was functionally involved in the nucleolar stress response associated with tRF5‐GlyTCC. DLD1 and HCT116 cells were divided into four groups: NC, tRF5‐GlyTCC inhibitor, siRPL23, and siRPL23 + tRF5‐GlyTCC inhibitor. Immunofluorescence analysis of nucleophosmin (NPM1) showed that RPL23 silencing resulted in marked redistribution of NPM1 from the nucleoli to a more dispersed nucleoplasmic pattern in both DLD1 and HCT116 cells (Figure 5A). In contrast, inhibition of tRF5‐GlyTCC alone did not induce an obvious nucleolar stress phenotype. Notably, simultaneous inhibition of tRF5‐GlyTCC did not substantially reverse the NPM1 redistribution induced by RPL23 depletion in either cell line. These findings indicate that RPL23 depletion is sufficient to induce an NPM1 redistribution phenotype consistent with nucleolar stress, and that this phenotype was not substantially altered by concomitant inhibition of tRF5‐GlyTCC.

**FIGURE 5:**
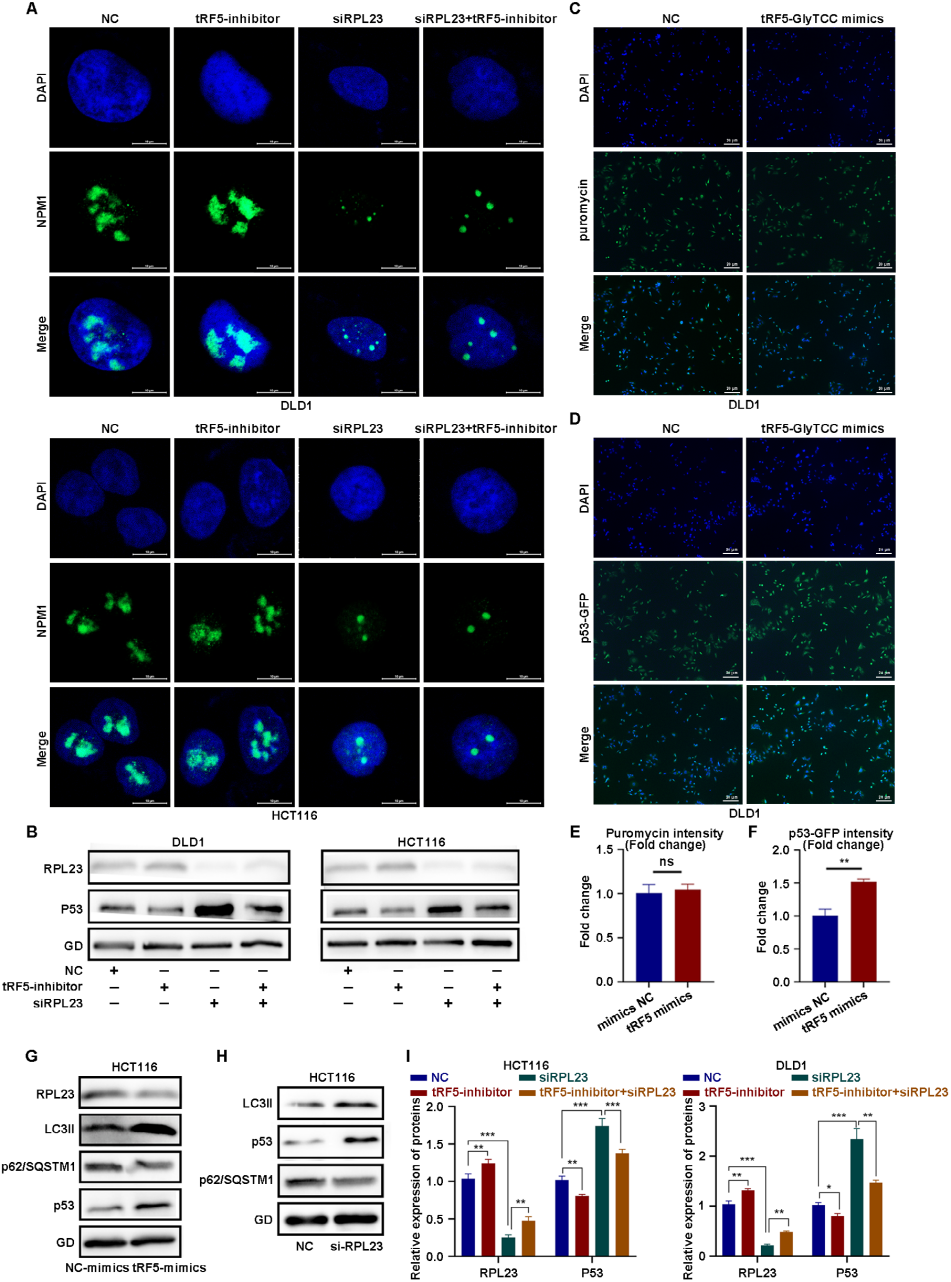
Further mechanistic insights into RPL23‐associated nucleolar stress and p53 signaling in CRC cells. (A)Immunofluorescence analysis of nucleophosmin (NPM1) in DLD1 and HCT116 cells transfected with NC, tRF5‐GlyTCC inhibitor, siRPL23, or siRPL23 + tRF5‐GlyTCC inhibitor. Scale bars: 10 µm. (B) Western blotting analysis of RPL23 and p53 in DLD1 and HCT116 cells following the indicated genetic manipulations. (C) SUnSET assay assessing global protein synthesis in DLD1 cells transfected with tRF5‐GlyTCC mimic or NC mimic. Puromycin incorporation indicates nascent protein synthesis. (D) Representative images of p53‐GFP reporter fluorescence in DLD1 cells following tRF5‐GlyTCC overexpression. (E–F) Quantification of puromycin incorporation (E) and p53‐GFP fluorescence intensity (F). (G–H) Western blotting analysis of RPL23, p53, LC3II, and p62/SQSTM1 expression in HCT116 cells following tRF5‐GlyTCC overexpression (G) or RPL23 knockdown (H). (I) Quantification of RPL23 and p53 protein levels shown in (B). Data are presented as mean ± SEM. ns, not significant; *P* < 0.05, ^*^ *P* < 0.01, ^**^ *P* < 0.001.

We next examined p53 signaling in this genetic context by Western blotting. RPL23 knockdown markedly reduced RPL23 protein levels and increased p53 abundance in both DLD1 and HCT116 cells. The increase in p53 was maintained following co‐transfection with siRPL23 and the tRF5‐GlyTCC inhibitor (Figure 5B,I). Together with the NPM1 redistribution phenotype, these findings support an association between RPL23 depletion, nucleolar stress, and increased p53 signaling.

We then investigated whether tRF5‐GlyTCC overexpression affected global protein synthesis. SUnSET analysis showed no significant change in puromycin incorporation following tRF5‐GlyTCC overexpression (Figure 5C,E), suggesting that global protein synthesis was not markedly altered under these experimental conditions.

Given the involvement of p53 signaling in the observed phenotype, we further assessed p53 pathway activity using a p53‐GFP reporter system. tRF5‐GlyTCC overexpression significantly increased p53‐GFP reporter fluorescence (Figure 5D,F), providing additional evidence of increased p53 pathway activity following tRF5‐GlyTCC restoration.

To further examine the relationship between RPL23 and autophagy‐associated signaling, Western blot analysis was performed in HCT116 cells. tRF5‐GlyTCC overexpression reduced RPL23 levels and was accompanied by increased p53 and LC3II levels and decreased p62 levels (Figure 5G; Figure S5A). Similarly, RPL23 knockdown increased p53 and LC3II levels and decreased p62 levels (Figure 5H; Figure S5B).

Collectively, these findings support a regulatory relationship in which tRF5‐GlyTCC modulates RPL23 expression, while RPL23 depletion is associated with nucleolar stress and increased p53‐related signaling without a detectable reduction in global protein synthesis.

### 3.6 RPL23 is Associated with Malignant Phenotypes and Contributes to the Effects of tRF5‐GlyTCC

To further clarify the functional significance of RPL23 in CRC, we first examined its expression in CRC cell lines. Both RT‐qPCR and Western blotting confirmed that RPL23 was highly expressed in CRC cell lines (Figure 6A; Figure S6A). siRNA‐mediated RPL23 silencing was confirmed by RT‐qPCR and Western blotting (Figure 6B; Figure S6B) and significantly inhibited cell proliferation, colony formation, and migration, as assessed by CCK‐8, colony formation, and Transwell assays (Figure 6C–G). These findings support an association between elevated RPL23 expression and malignant CRC phenotypes.

**FIGURE 6:**
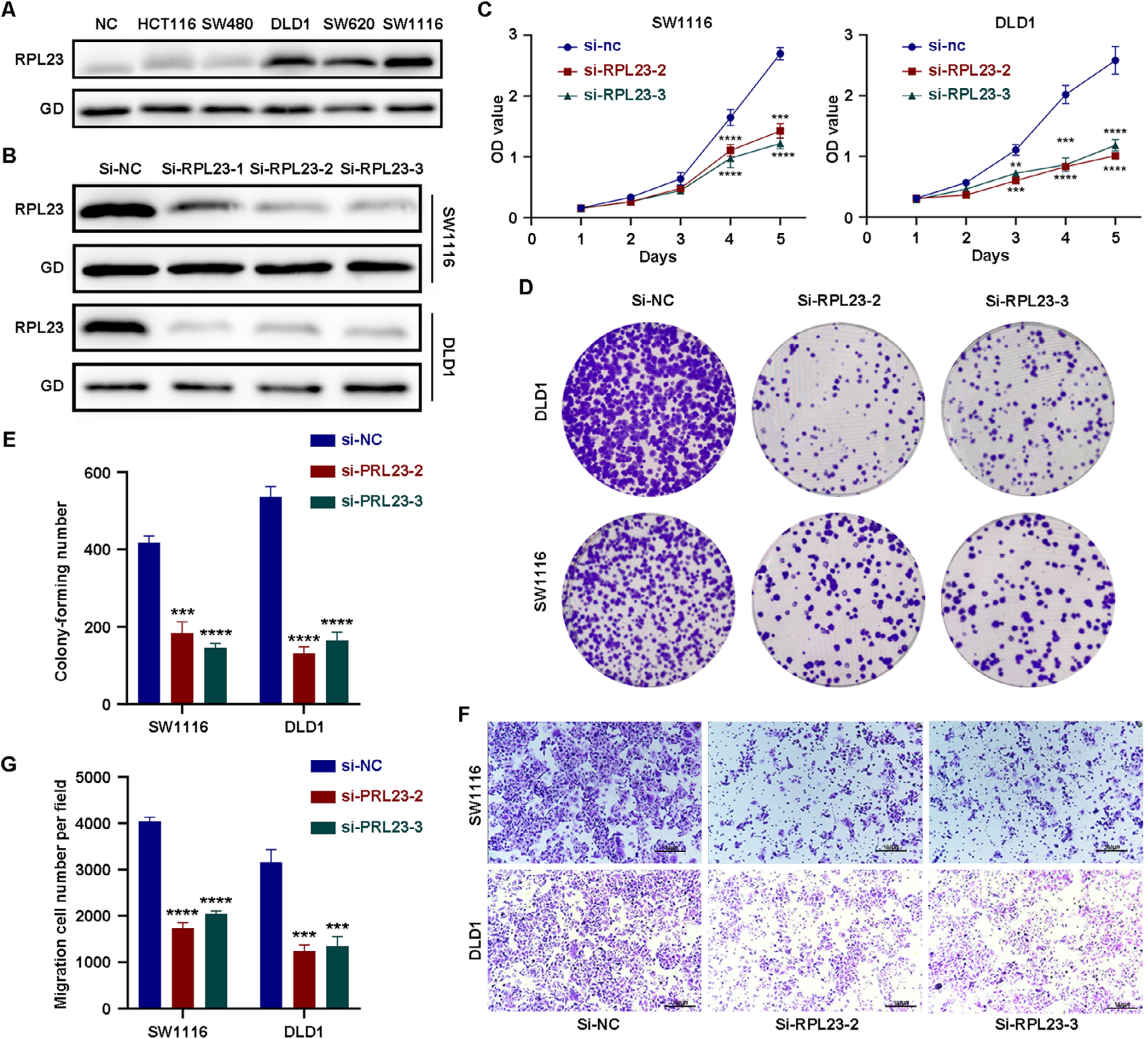
Functional analysis of RPL23 in colorectal cancer cells. (A)RPL23 expression levels in CRC cell lines determined by Western blotting. (B) Knockdown efficiency of RPL23 siRNA assessed by RT‐qPCR and Western blotting. (C) Cell viability assessed by CCK‐8 assays following RPL23 silencing. (D‐E) Colony formation assays following RPL23 knockdown. (F–G) Transwell migration assays evaluating cell migration following RPL23 silencing. *P* < 0.05, ^*^ *P* < 0.01, ^**^ *P* < 0.001, ^***^ *P* < 0.0001.

To determine whether RPL23 contributes to the tumor‐suppressive effects of tRF5‐GlyTCC, genetic interaction experiments were performed. Co‐transfection of tRF5‐GlyTCC inhibitor with siRPL23 attenuated the increased proliferation and migration associated with tRF5‐GlyTCC inhibition (Figure 7A–G). This partial attenuation supports the involvement of RPL23 in the effects of tRF5‐GlyTCC on CRC cell proliferation and migration.

**FIGURE 7:**
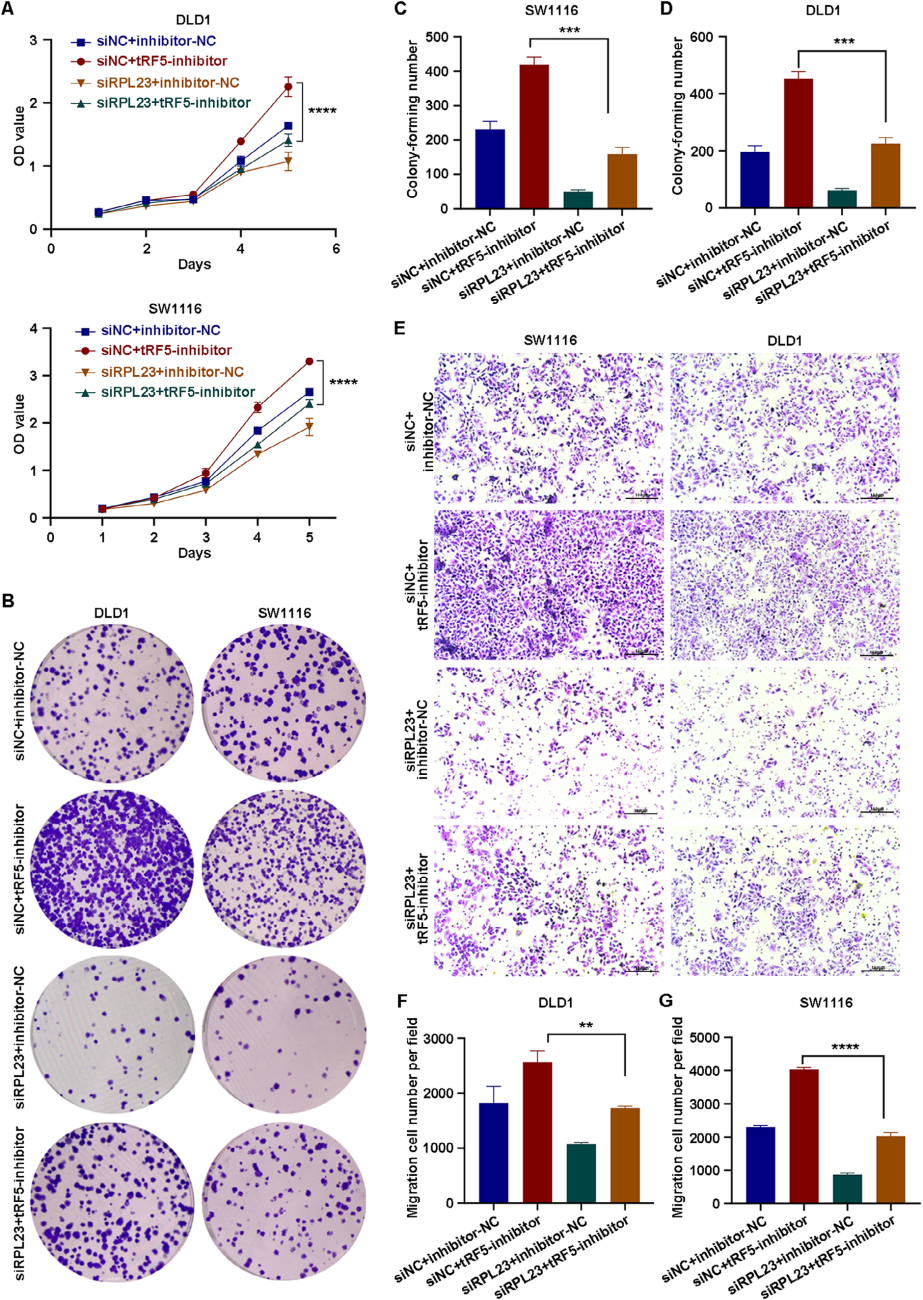
RPL23 contributes to the effects of tRF5‐GlyTCC on CRC cell phenotypes. (A–G) CCK‐8, colony formation, and Transwell migration assays performed in CRC cells co‐transfected with tRF5‐GlyTCC inhibitor and siRPL23, as indicated. *P* < 0.05, ^*^ *P* < 0.01, ^**^ *P* < 0.001, ^***^ *P* < 0.0001.

### 3.7 Overexpression of tRF5‐GlyTCC Suppresses Tumor Growth and Enhances CRC Sensitivity to 5‐FU In Vivo

Given the associations of tRF5‐GlyTCC with RPL23, p53 signaling, and autophagy, we next investigated its potential relevance to chemotherapy response. CCK‐8 assays showed that tRF5‐GlyTCC overexpression increased the sensitivity of DLD1, HCT116, and SW1116 cells to 5‐fluorouracil (5‐FU), as reflected by reduced 5‐FU IC_50_ values (Figure 8A–C; Figure S7A,B; Table S3).

**FIGURE 8:**
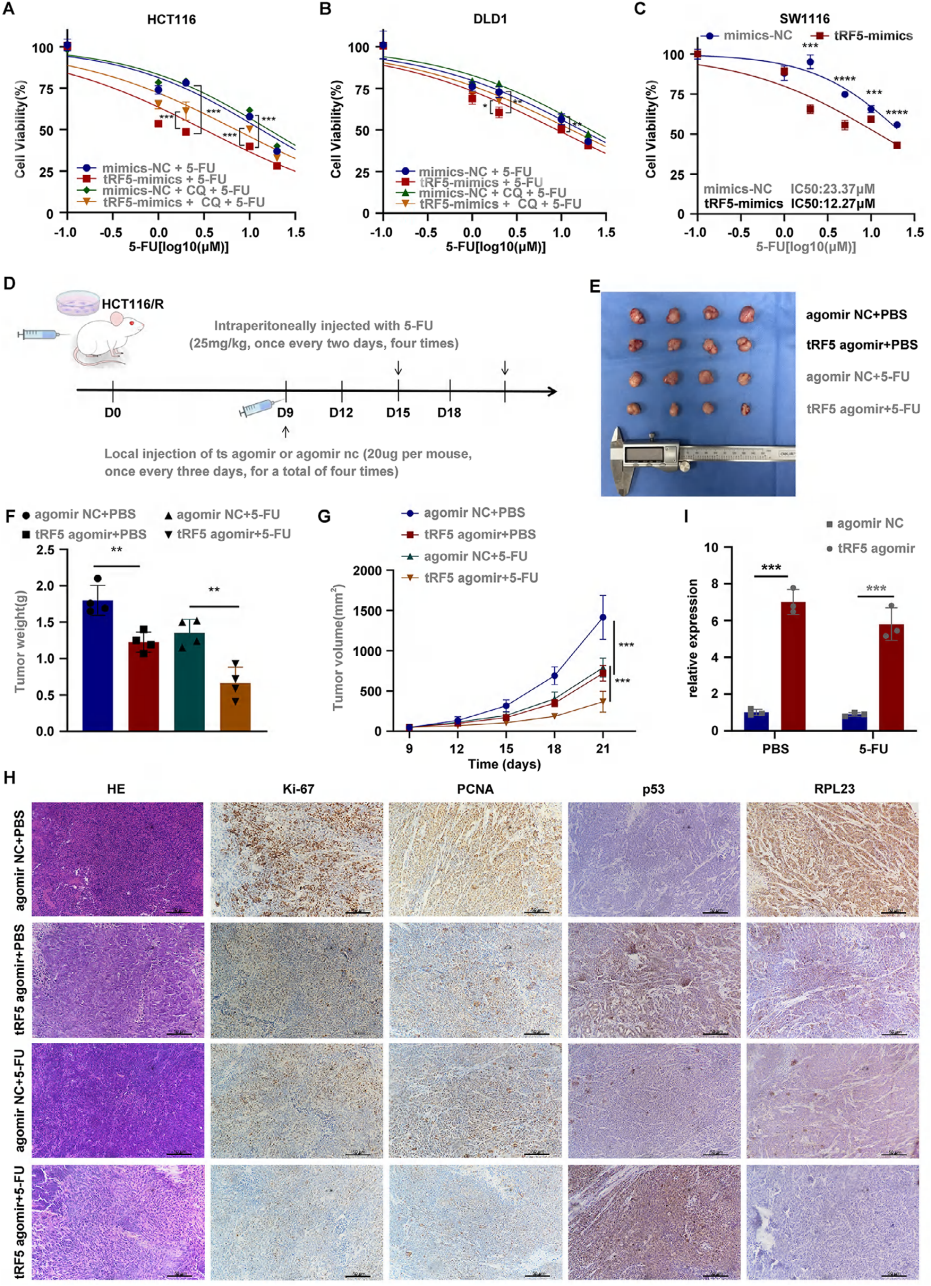
Effects of tRF5‐GlyTCC on tumor growth and response to 5‐FU. (A–B) CCK‐8‐based 5‐FU dose–response assays in HCT116 and DLD1 cells transfected with NC mimic or tRF5‐GlyTCC mimic and treated with increasing concentrations of 5‐FU in the presence or absence of chloroquine (CQ). (C) CCK‐8‐based 5‐FU dose‐response assay in SW1116 cells transfected with NC mimic or tRF5‐GlyTCC mimic. (D–G) Tumor growth curves and tumor weights in HCT116 xenograft models treated with agomir NC + PBS, tRF5‐GlyTCC agomir + PBS, agomir NC + 5‐FU, or tRF5‐GlyTCC agomir + 5‐FU. (H) Representative H&E staining and immunohistochemical staining for Ki‐67, PCNA, p53, and RPL23 in tumor tissue sections from each treatment group. Ki‐67 and PCNA show nuclear localization, with changes consistent with differences in tumor cell proliferation among the treatment groups. (I) RT‐qPCR analysis of tRF5‐GlyTCC abundance in xenograft tumor tissues following agomir treatment. Data are presented as mean ± SD. Statistical significance for the 5‐FU dose–response and CQ experiments was determined using two‐way ANOVA followed by Šídák's multiple‐comparisons test. *P* < 0.05, ^*^ *P* < 0.01, ^**^ *P* < 0.001, ^***^ *P* < 0.0001.

To determine whether autophagy contributes functionally to the enhanced 5‐FU sensitivity associated with tRF5‐GlyTCC, we performed pharmacological inhibition experiments using chloroquine (CQ). HCT116 and DLD1 cells transfected with NC mimic or tRF5‐GlyTCC mimic were treated with increasing concentrations of 5‐FU in the presence or absence of CQ, followed by CCK‐8 analysis (Figure 8A,B). Consistent with the initial observations, tRF5‐GlyTCC overexpression markedly sensitized both HCT116 and DLD1 cells to 5‐FU. In HCT116 cells, the 5‐FU IC_50_ decreased from 9.660 µm in the NC mimic group to 1.922 µm following tRF5‐GlyTCC overexpression. CQ treatment increased the IC_50_ to 4.573 µm in tRF5‐GlyTCC mimic‐treated cells. Similarly, in DLD1 cells, tRF5‐GlyTCC overexpression decreased the 5‐FU IC_50_ from 13.51 to 6.241 µm, whereas CQ treatment increased the IC_50_ to 9.637 µm (Table S3). These findings indicate that pharmacological inhibition of autophagy partially attenuated the enhanced 5‐FU sensitivity associated with tRF5‐GlyTCC overexpression, supporting a functional contribution of autophagy to tRF5‐GlyTCC‐mediated 5‐FU sensitization.

We next evaluated the therapeutic relevance of tRF5‐GlyTCC in vivo using an HCT116 xenograft model. Intratumoral administration of tRF5‐GlyTCC agomir significantly suppressed tumor growth compared with the agomir NC group, whereas the combination of tRF5‐GlyTCC agomir and 5‐FU produced a more pronounced reduction in tumor volume and weight (Figure 8D–G). Immunohistochemical analysis showed that the combination treatment group exhibited the lowest Ki‐67, PCNA, and RPL23 expression and the highest p53 expression (Figure 8H), consistent with the observed antitumor effects.

To further verify the in vivo activity of tRF5‐GlyTCC agomir, tRF5‐GlyTCC abundance was quantified in xenograft tumor tissues by RT‐qPCR. tRF5‐GlyTCC levels were significantly increased in tumors treated with tRF5‐GlyTCC agomir compared with the corresponding agomir NC groups under both PBS and 5‐FU treatment conditions. Two‐way ANOVA showed a significant main effect of tRF5‐GlyTCC overexpression (*P* < 0.001), whereas neither 5‐FU treatment nor the interaction between the two factors showed a significant effect. These findings demonstrate that intratumoral administration of tRF5‐GlyTCC agomir increased tRF5‐GlyTCC abundance in xenograft tumor tissues (Figure 8I).

Collectively, these findings demonstrate that tRF5‐GlyTCC enhances CRC sensitivity to 5‐FU, at least in part through autophagy‐associated mechanisms, and suppresses tumor growth in vivo. These results provide proof‐of‐concept evidence supporting the potential of tRF5‐GlyTCC as a sensitizing strategy for 5‐FU‐based treatment.

### 3.8 NSUN3‐Mediated m5C Modification Contributes to the Stability of tRF5‐GlyTCC

To identify upstream regulators associated with the downregulation and stability of tRF5‐GlyTCC, we investigated RNA modifications that may contribute to its regulation. Multiple potential m5C modification sites within the annotated precursor tRNA‐Gly‐TCC were predicted using an online prediction tool (Figure S8A). Among these candidate sites, nucleotide C66 exhibited the highest Random Forest probability score and was therefore selected for further investigation. m5C MeRIP‐qPCR demonstrated significant m5C enrichment in the tRNA‐Gly‐TCC region encompassing C66 (Figure 9A,B).

**FIGURE 9:**
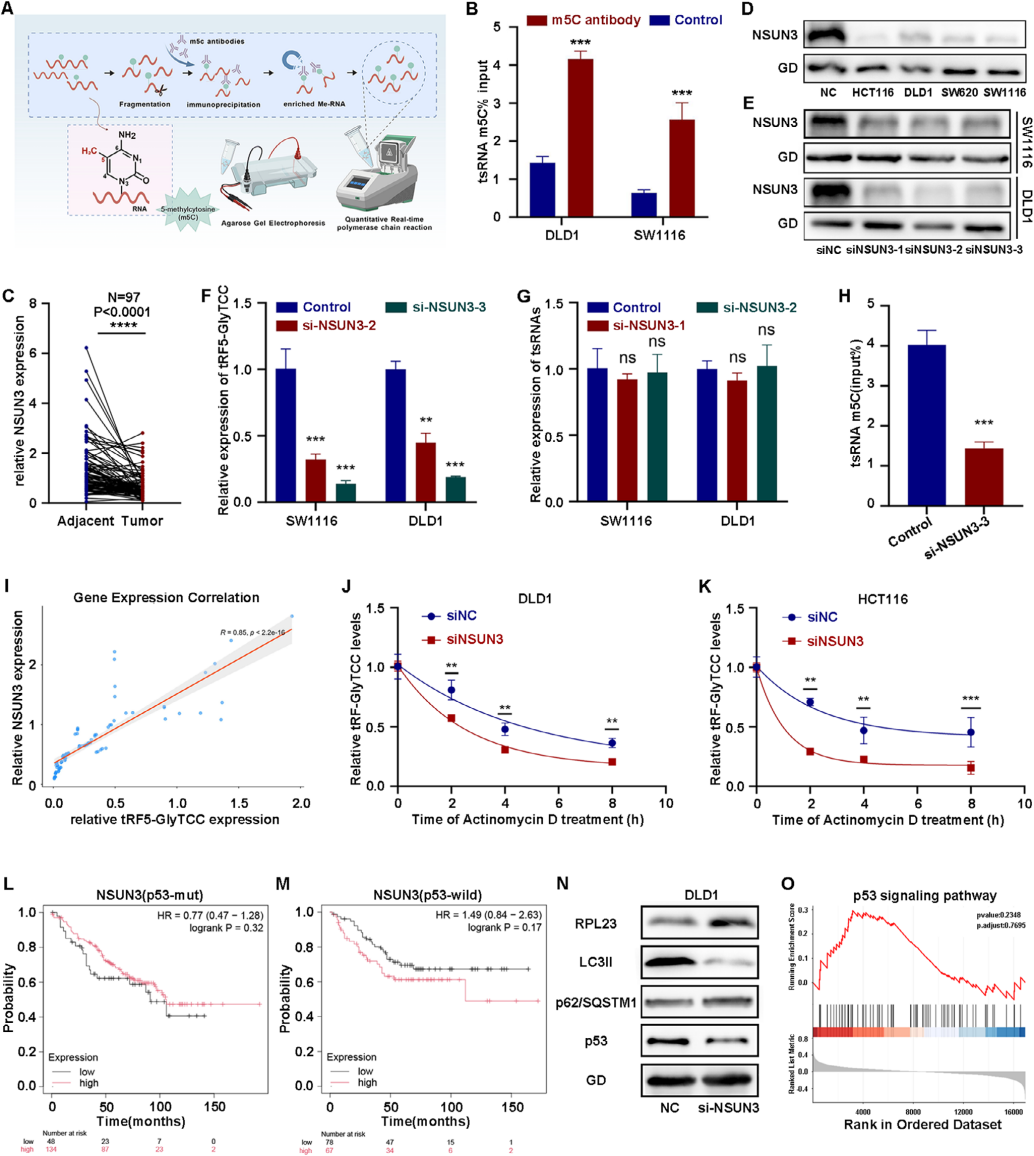
NSUN3‐associated m5C modification contributes to the stability of tRF5‐GlyTCC. (A–B) Prediction and MeRIP‐qPCR validation of m5C modification in the annotated precursor tRNA‐Gly‐TCC region encompassing the predicted C66 site. (C–D) NSUN3 expression in CRC tissues (*n* = 97) and CRC cell lines. (E) Knockdown efficiency of NSUN3 siRNA in CRC cells. (F) Effects of NSUN3 silencing on tRF5‐GlyTCC expression. (G) Effects of NSUN3 silencing on the abundance of the annotated precursor tRNA‐Gly‐TCC. (H) MeRIP‐qPCR analysis of m5C enrichment in the precursor tRNA‐Gly‐TCC region encompassing C66 following NSUN3 knockdown. (I) Pearson correlation analysis between NSUN3 and tRF5‐GlyTCC expression in 97 paired CRC tissue samples (*n* = 97). (J–K) Actinomycin D chase assays showing tRF5‐GlyTCC decay in DLD1 and HCT116 cells following NSUN3 knockdown. tRF5‐GlyTCC levels were measured by RT‐qPCR at 0, 2, 4, and 8 h after Actinomycin D treatment and normalized to the corresponding 0 h level. Decay curves were fitted by nonlinear regression, and statistical significance was determined using two‐way ANOVA followed by Šídák's multiple‐comparisons test. (L–M) Kaplan–Meier survival analysis of the prognostic relevance of NSUN3 stratified by TP53 mutation status using the Kaplan–Meier Plotter platform. (L) TP53‐mutant; (M) TP53‐wild‐type. (N) Western blotting analysis showing that NSUN3 knockdown increases RPL23 expression and is accompanied by decreased p53 and LC3II levels and increased p62/SQSTM1 levels. (O) Gene set enrichment analysis (GSEA) showing positive associations between NSUN3 expression and p53‐related signaling pathways. *P* < 0.05, ^*^ *P* < 0.01, ^**^ *P* < 0.001, ^***^ *P* < 0.0001.

We next investigated the potential involvement of NSUN3 in the regulation of tRF5‐GlyTCC. Consistent with the TCGA analysis, NSUN3 expression was significantly reduced in CRC tissues compared with matched adjacent non‐tumor tissues in our expanded cohort of 97 paired samples (Figure 9C; Figure S8B). NSUN3 expression was also lower in CRC cell lines than in NCM460 cells (Figure 9D). Silencing NSUN3 in CRC cells (Figure 9E) markedly reduced tRF5‐GlyTCC expression (Figure 9F).

To determine whether the reduction in tRF5‐GlyTCC was attributable to altered precursor abundance, we quantified the annotated precursor tRNA‐Gly‐TCC. NSUN3 depletion did not significantly affect precursor tRNA‐Gly‐TCC abundance in either SW1116 or DLD1 cells (Figure 9G), suggesting that the reduction in mature tRF5‐GlyTCC was unlikely to be explained simply by decreased precursor availability. In contrast, NSUN3 knockdown significantly reduced m5C enrichment in the tRNA‐Gly‐TCC region encompassing C66 (Figure 9H), supporting an association between NSUN3 and m5C modification of the precursor tRNA‐Gly‐TCC.

We next examined the relationship between NSUN3 and tRF5‐GlyTCC in clinical samples. Pearson correlation analysis using all 97 paired CRC tissues showed a strong positive correlation between NSUN3 and tRF5‐GlyTCC expression (Figure 9I), further supporting their coordinated regulation in CRC.

To determine whether NSUN3 affects tRF5‐GlyTCC stability, we performed an Actinomycin D chase assay in DLD1 and HCT116 cells. Following transcriptional inhibition with Actinomycin D, tRF5‐GlyTCC levels were monitored at 0, 2, 4, and 8 h by RT‐qPCR and normalized to the corresponding 0 h values. Compared with control cells, NSUN3 knockdown markedly accelerated tRF5‐GlyTCC decay in both DLD1 and HCT116 cells (Figure 9J,K). Nonlinear regression analysis showed that the half‐life of tRF5‐GlyTCC decreased from 3.31 to 1.78 h in DLD1 cells and from 1.66 to 0.70 h in HCT116 cells following NSUN3 knockdown (Table S4). These findings provide direct evidence that NSUN3 contributes to the stability of tRF5‐GlyTCC.

We further investigated the clinical relevance of NSUN3 expression. Survival analysis using the Kaplan–Meier Plotter platform showed that the prognostic association of NSUN3 differed according to TP53 mutation status (Figure 9L,M), suggesting that the clinical significance of NSUN3 may vary according to the p53 signaling context.

Finally, we examined whether NSUN3 depletion was associated with changes in RPL23, p53, and autophagy‐associated proteins. NSUN3 knockdown increased RPL23 expression and was accompanied by reduced p53 and LC3II levels and increased p62/SQSTM1 levels (Figure 9N; Figure S8C), consistent with the changes observed following tRF5‐GlyTCC modulation. These findings suggest that NSUN3 may be associated with RPL23‐linked p53 signaling and autophagy‐related responses, potentially through its regulation of tRF5‐GlyTCC. GSEA further showed that NSUN3 expression was positively associated with p53‐related signaling pathways (Figure 9O).

Collectively, these findings suggest that NSUN3‐mediated m5C modification contributes to the stability of tRF5‐GlyTCC and is associated with RPL23 expression and p53‐related cellular responses, including autophagy, in CRC.

## 4 Discussion

In this study, we identify tRF5‐GlyTCC as a previously uncharacterized tumor‐suppressive tsRNA in colorectal cancer (CRC). tRF5‐GlyTCC was significantly downregulated in CRC tissues and cell lines, and its reduced expression was associated with aggressive clinicopathological features and poor overall survival. Functional studies consistently showed that restoration of tRF5‐GlyTCC suppressed CRC cell proliferation and migration in vitro and inhibited tumor growth in vivo, supporting a tumor‐suppressive regulatory role for this tsRNA. Recent studies further support the functional involvement of tRNA‐derived fragments in cellular stress responses, showing that stress‐responsive tRNA cleavage can generate biologically active tRNA‐derived species with context‐dependent effects on cancer cells [28].

Mechanistically, our findings identify RPL23 as a direct target of tRF5‐GlyTCC. The interaction between tRF5‐GlyTCC and the RPL23 3′UTR was supported by integrated target prediction, reporter assays, and reciprocal changes in RPL23 expression following tRF5‐GlyTCC modulation. Genetic perturbation experiments further indicated that RPL23 contributes to the biological effects of tRF5‐GlyTCC. RPL23 depletion was accompanied by redistribution of NPM1 from the nucleoli to the nucleoplasm, a phenotype consistent with nucleolar stress, together with increased p53 signaling. These findings extend previous observations that RPL23 can participate in p53 regulation, including through interactions with the MDM2–p53 regulatory system, and suggest that perturbation of RPL23 may engage nucleolar stress‐associated p53 responses in CRC cells. Importantly, our genetic interaction experiments and NPM1 redistribution analyses support an association between RPL23 perturbation and nucleolar stress, but do not establish nucleolar stress as an obligatory intermediate for p53 activation. The context‐dependent effects of RPL23 may therefore reflect differences in the cellular stress‐response programs engaged following perturbation of ribosomal homeostasis.

Consistent with this interpretation, RPL23 perturbation did not produce a detectable reduction in global protein synthesis under the conditions tested, as assessed by SUnSET analysis. This finding argues against a nonspecific translational shutdown as the primary explanation for the observed p53 response and is compatible with the involvement of nucleolar stress‐related processes. Downstream, tRF5‐GlyTCC restoration was associated with increased p53 signaling and autophagy‐related changes, including increased LC3‐II and ATG5 levels, reduced p62 levels, and increased autophagosome formation observed by transmission electron microscopy. The additional accumulation of LC3‐II following chloroquine treatment, together with attenuation of p62 reduction, further supported enhanced autophagic flux under the experimental conditions examined. Similar RPL23‐associated p53 and autophagy‐related responses were also observed in HCT116 cells with wild‐type p53, supporting the reproducibility of these findings across CRC cellular contexts. Nevertheless, the present data support an association between p53 signaling and autophagy rather than establishing a strictly linear or p53‐dependent pathway.

Autophagy‐associated activity also appears to contribute to the enhanced sensitivity of CRC cells to 5‐fluorouracil (5‐FU) following tRF5‐GlyTCC restoration. Autophagy can exert context‐dependent effects on tumor responses to chemotherapy, functioning either as a cytoprotective mechanism or as a component of treatment‐associated cellular stress [29, 30]. In our experimental system, pharmacological inhibition with chloroquine partially attenuated the 5‐FU‐sensitizing effect of tRF5‐GlyTCC, supporting a functional contribution of autophagy‐associated processes to this phenotype. Thus, tRF5‐GlyTCC may link RPL23‐associated stress responses with autophagy‐related cellular responses that contribute to chemotherapy sensitivity. Further studies using complementary genetic and pharmacological approaches will be required to define the precise contribution and molecular dependence of autophagy in this process.

From a translational perspective, our in vivo findings provide proof‐of‐concept evidence that restoration of tRF5‐GlyTCC can suppress CRC tumor growth and enhance the antitumor effect of 5‐FU. Intratumoral administration of tRF5‐GlyTCC agomir increased tRF5‐GlyTCC abundance in xenograft tumor tissues and was associated with reduced tumor growth, particularly in combination with 5‐FU. However, this delivery strategy has important limitations for clinical translation. Although intratumoral administration demonstrates local biological activity, it does not establish the feasibility of systemic delivery, tumor‐specific biodistribution, or spatial uptake of the agomir. The current RT‐qPCR analysis confirms increased tRF5‐GlyTCC abundance in tumor tissue but does not provide information on its cellular or intratumoral distribution. These limitations highlight the need for future studies evaluating more clinically relevant delivery systems that improve RNA stability, tumor targeting, cellular uptake, and intracellular release, together with systematic assessment of pharmacokinetics, biodistribution, and safety.

RNA m5C modification has increasingly been implicated in gastrointestinal tumor biology, with recent studies highlighting its roles in RNA stability, tRNA regulation, and tumor progression [31]. In this context, our findings identify NSUN3‐associated m5C modification as an upstream regulatory mechanism that contributes to the stability of tRF5‐GlyTCC. NSUN3 expression was reduced in CRC tissues and cell lines and positively correlated with tRF5‐GlyTCC expression in the expanded clinical cohort. NSUN3 depletion reduced mature tRF5‐GlyTCC abundance without significantly altering the abundance of the annotated precursor tRNA‐Gly‐TCC. In parallel, m5C MeRIP‐qPCR showed reduced m5C enrichment in the precursor tRNA‐Gly‐TCC region encompassing C66 following NSUN3 knockdown. Importantly, Actinomycin D chase experiments demonstrated accelerated decay and a reduced half‐life of tRF5‐GlyTCC after NSUN3 depletion in both DLD1 and HCT116 cells. Together, these findings support a role for NSUN3‐associated m5C modification in maintaining the stability of mature tRF5‐GlyTCC.

The unchanged precursor tRNA‐Gly‐TCC abundance following NSUN3 depletion further suggests that the reduction in mature tRF5‐GlyTCC is not simply attributable to decreased precursor availability. However, tRF abundance can be influenced by multiple steps of tRNA metabolism, including precursor processing and cleavage of mature tRNA [32, 33]. Therefore, altered processing efficiency or other RNA metabolic mechanisms may also contribute to tRF5‐GlyTCC regulation. Although our data support an association between NSUN3 and m5C modification within the precursor tRNA‐Gly‐TCC region, they do not establish that NSUN3 directly methylates C66 or determine whether NSUN3 regulates tRF5‐GlyTCC biogenesis exclusively through this modification. Direct biochemical validation and further dissection of tRNA processing will be required to clarify the precise molecular mechanism.

The relationship between NSUN3 and the downstream regulatory network provides additional support for the proposed model. NSUN3 depletion increased RPL23 expression and produced changes in p53 and autophagy‐related markers that broadly resembled those observed following tRF5‐GlyTCC inhibition. Together with the positive correlation between NSUN3 and tRF5‐GlyTCC in clinical samples, these findings are consistent with NSUN3 influencing the RPL23‐associated p53 and autophagy‐related network, at least in part through regulation of tRF5‐GlyTCC. The differential prognostic association of NSUN3 according to TP53 mutation status further suggests that its biological and clinical relevance may depend on the underlying p53 signaling context. However, these observations do not establish a direct NSUN3–tRF5‐GlyTCC–RPL23 signaling cascade.

Several limitations should be acknowledged. First, although the current data provide convergent evidence linking RPL23 perturbation with nucleolar stress‐associated p53 responses, they do not establish nucleolar stress as a required intermediate for p53 activation, and a definitive siRNA‐resistant RPL23 rescue experiment was not performed. More direct manipulation of nucleolar stress signaling and RPL23 rescue approaches will therefore be important to further define the causal hierarchy of this regulatory relationship. Second, additional targets of tRF5‐GlyTCC may exist and could contribute to its biological effects, while the influence of tumor microenvironmental factors on tsRNA regulation and function remains unexplored. Third, although intratumoral agomir administration increased tRF5‐GlyTCC levels in xenograft tumors, circulating tRF5‐GlyTCC levels, systemic biodistribution, and spatial tissue distribution were not assessed, and the current model does not establish the feasibility of systemic therapeutic delivery. Fourth, the present study primarily addresses 5‐FU sensitivity rather than acquired 5‐FU resistance; whether tRF5‐GlyTCC is altered during resistance development or can overcome established resistance remains to be determined. Finally, larger and independent clinical cohorts will be needed to further validate the diagnostic and prognostic significance of tRF5‐GlyTCC and to determine its clinical utility across different patient populations.

Taken together, our findings support a working model in which NSUN3‐associated m5C modification contributes to tRF5‐GlyTCC stability, whereas reduced tRF5‐GlyTCC expression is associated with increased RPL23 abundance. RPL23 perturbation is accompanied by NPM1 redistribution, p53 signaling, and autophagy‐associated responses (Figure 10). Although these relationships should not be interpreted as a strictly linear causal pathway, this model provides a framework linking tsRNA stability, nucleolar stress responses, and chemotherapy sensitivity in CRC.

**FIGURE 10:**
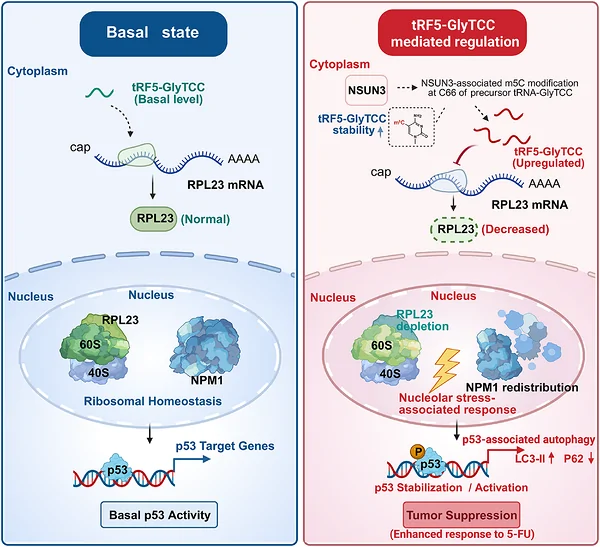
Proposed working model of NSUN3‐associated regulation of tRF5‐GlyTCC in colorectal cancer. The proposed model summarizes the relationships supported by the present study. NSUN3‐associated m5C modification in the annotated precursor tRNA‐Gly‐TCC region, encompassing C66, is associated with maintenance of tRF5‐GlyTCC stability. Increased tRF5‐GlyTCC expression is associated with reduced RPL23 expression, which is accompanied by NPM1 redistribution and a nucleolar stress phenotype, together with increased p53 signaling and autophagy‐associated responses. These changes are associated with tumor‐suppressive effects and enhanced sensitivity to 5‐fluorouracil (5‐FU). The model is intended to represent the experimentally supported relationships and does not imply a strictly linear causal pathway.

In summary, we identify tRF5‐GlyTCC as a tumor‐suppressive tsRNA that is downregulated in CRC and associated with aggressive disease features. Our findings indicate that tRF5‐GlyTCC directly regulates RPL23 and is associated with nucleolar stress‐related p53 signaling and autophagy‐associated responses, thereby contributing to CRC progression and 5‐FU sensitivity. At the upstream level, NSUN3‐associated m5C modification contributes to the stability of tRF5‐GlyTCC, providing an additional layer of epitranscriptomic regulation. Together, these findings link tsRNA regulation with ribosomal and nucleolar stress responses and chemotherapy sensitivity, and provide proof‐of‐concept support for further investigation of tRF5‐GlyTCC as a potential therapeutic target in CRC.

## Author Contributions

X.L. Conceptualization, Data curation, Formal analysis, Investigation, Methodology, Validation – Verification, Writing – original draft – Preparation. C.T., F.S. and Y.W. Data curation, Formal analysis, Investigation, Validation – Verification, Writing – original draft – Preparation. Y.C. Data curation, Formal analysis, Investigation, Writing – review & editing – Preparation. S.L. and Z.Y. Data curation, Formal analysis, Validation – Verification, Writing – review & editing – Preparation. G.W. Resources, Supervision, Writing – review & editing – Preparation. X.W. Project administration, Resources, Supervision, Writing – review & editing – Preparation. All authors reviewed the manuscript and approved the final version.

## Funding

This study was supported by the National Natural Science Foundation of China (81972015), General Project of the Health Commission of Jiangsu Province (H2018074), Nantong Science and Technology Project (JC2019121). This study was supported by a grant from Jiangsu Provincial Research Hospital (YJXYY202204‐YSB72).

## Ethics Statement

Clinical samples were collected after informed consent was obtained from the subjects, and studies involving human subjects were reviewed and approved by the Ethics Committee of the Affiliated Hospital of Nantong University(2019‐L053). The experiments were approved by the Animal Experiment Ethics Review Form of Nantong University Animal Experiment Center (Approval No. S20250821‐001).

## Conflicts of Interest

The authors declare no conflicts of interest.

## Supporting information




**Supporting File 1**: advs78345‐sup‐0001‐SuppMat.docx.

**Supporting File 2**: advs78345‐sup‐0002‐TableS2.xlsx.

## Data Availability

The data supporting the findings of this study can be accessed upon reasonable request to the corresponding author.
