# Supplementary material for: NSUN3‐Associated m5C Modification of tRF5‐GlyTCC Suppresses Colorectal Cancer Progression via RPL23‐Linked Nucleolar Stress and p53‐Associated Autophagy and Enhances 5‐FU Sensitivity
Source: Adv Sci (Weinh). 2026 Oct 22:e78345. Online ahead of print. doi: 10.1002/advs.78345 (PMC13691228; doi:10.1002/advs.78345)
Supplement: Supplementary file 1 [file ADVS-9999-e78345-s002.docx]

**Supplementary Information**

**Supplementary Figure 1.**

(A) Sanger sequencing confirming the nucleotide sequence of tRF5-GlyTCC (5′-GCGTTGGTGGTATAGTGGTGAGC-3′). (B) Relative expression of tRF5-GlyTCC in CRC cell lines determined by RT-qPCR.

A

BA

**Supplementary Figure 2.**

(A, B) Knockdown and overexpression efficiency of tRF5-GlyTCC in CRC cells, as assessed by RT-qPCR.

C

**Supplementary Figure 3.**

(A) Schematic illustration of wild-type (WT) and mutant (MUT) NACC1 3′-UTR luciferase reporter constructs.(B) Dual-luciferase reporter assays in SW1116 cells co-transfected with tRF5-GlyTCC mimics and WT or MUT reporters.(C) RPL23 expression in colorectal cancer based on TCGA database analysis.(D) Gene set enrichment analysis (GSEA) of 50 hallmark pathways associated with RPL23 expression across multiple cancer types.

**Supplementary Figure 4.**

(A, B) Quantification of protein expression levels shown in Figure 4C.

**Supplementary Figure 5.**

(A) Quantification of protein expression levels shown in Figure 5G. (B) Quantification of protein expression levels shown in Figure 5H.

NSUN3

**Supplementary Figure 6.**

(A) Relative RPL23 expression levels in CRC cell lines measured by RT-qPCR. (B) Validation of RPL23 knockdown efficiency in CRC cells by RT-qPCR.

**Supplementary Figure 7.**

(A) Cell viability of DLD1 cells treated with increasing concentrations of 5-FU following transfection with NC mimic or tRF5-GlyTCC mimic. (B) Cell viability of HCT116 cells treated with increasing concentrations of 5-FU following transfection with NC mimic or tRF5-GlyTCC mimic.

**Supplementary Figure 8.**

(A) Predicted m5C modification sites within tRF5-GlyTCC identified using the RNA m5C Finder. (B) NSUN3 expression levels in colorectal cancer derived from TCGA database analysis. (C) Quantification of protein expression levels shown in Figure 9N.

**Supplementary Table 1 Primer sequence**

| **Gene name** | **Primer sequence** |
| --- | --- |
| tRF5-23-GlyTCC-2 ( tRF5-GlyTCC ) | F: GCGTTGGTGGTATAGT  R:GTCGTATCCAGTGCAGGGTCCGAGGT  RT:GTCGTATCCAGTGCAGGGTCCGAGGT  ATTCGCACTGGATACGACGCTCAC |
| tRF5-20-CysGCA-25 | F: GGGGTATATCTCAG  R:GTCGTATCCAGTGCAGGGTCCGAGGT  RT:GTCGTATCCAGTGCAGGGTCCGAGGT  ATTCGCACTGGATACGACGCTCAC |
| tRF5-30-LysCTT-2 | F:CCGGCTAGCTCAGTCG  R:GTCGTATCCAGTGCAGGGTCCGAGGT  RT:GTCGTATCCAGTGCAGGGTCCGAGGT  ATTCGCACTGGATACGACGCTCTA |
| precursor tRNA-GlyTCC | F:GCTGCCTTCCAAGCAGT  R:GTCGTATCCAGTGCAGGGTCCGAGGT  RT:GTCGTATCCAGTGCAGGGTCCGAGGT  ATTCGCACTGGATACGACGCTCAC |
| U6 | F: CTCGCTTCGGCAGCACATATACT  R: GTCGTATCCAGTGCAGGGTCCGAGGT  RT: AAAATATGGAACGCTTCACGAATTTG |
| tRF5-23-GlyTCC-2 mimics | sense:GCGUUGGUGGUAUAGUGGUGAGC  Antisense:UCACCACUAUACCACCAACGCUU |
| mimics NC | Sense: 5'-UUCUCCGAACGUGUCACGUTT-3’  Antisense: 5'-ACGUGACACGUUCGGAGAATT-3’ |
| tRF5-23-GlyTCC-2 inhibitor | sense:GCUCACCACUAUACCACCAACGC  Antisense: — |
| inhibitor N.C. | CAGUACUUUUGUGUAGUACAA |
| tRF5-23-GlyTCC-2 agomir | sense:GCGUUGGUGGUAUAGUGGUGAGC  Antisense:UCACCACUAUACCACCAACGCUU |
| Agomir NC | sense:UUCUCCGAACGUGUCACGUTT  Antisense:ACGUGACACGUUCGGAGAATT |
| GAPDH | F: GAGATCCCTCCAAAATCAAGTG  R: GAGTCCTTCCACGATACCAAAG |
| DDX5 | F:AAACCCTGGGGAGAAATTAGTT R:CAGTGGGTTCAGTGAAATTCTG |
| RPL23 | F:GTAGAAAAGATGGCGTGTTTCT  R:AGAACCTTTCATCTCGCCTTTA |
| NACC1 | F:CACACCTCTTTCACGCAGGACAG  R:ACGCCGCTCCTCTCCTTACAG |
| NSUN3 | F:ATGCTGTCCTGCTTAACCGATTC  R:TGAGAGAGTGTGTGATAGCCCTTC |
| precursor tRNA-GlyTCC-M5C | F:GCGTTGGTGGTATAGTGGTGAGCATAG  R:TGGTGCGTTGGCCGGGAATC |

Note: F, Forward primer; R, Reverse primer; RT, Reverse Transcription.

## ****Supplementary Table 3.**** IC50 values of 5-FU in CRC cells treated with tRF5-GlyTCC mimic in the presence or absence of chloroquine

| Cell line | Treatment | IC50 (μM) |
| --- | --- | --- |
| HCT116 | NC mimic | 9.660 |
|  | tRF5-GlyTCC mimic | 1.922 |
|  | NC mimic+CQ | 12.11 |
|  | tRF5-GlyTCC mimic+CQ | 4.573 |
| DLD1 | NC mimic | 13.51 |
|  | tRF5-GlyTCC mimic | 6.241 |
|  | NC mimic+CQ | 18.63 |
|  | tRF5-GlyTCC mimic+CQ | 9.637 |

## ****Supplementary Table 4.**** Nonlinear regression parameters for Actinomycin D-mediated decay of tRF5-GlyTCC.

| Cell line | Group | Half-life (h) | K (h⁻¹) | R² |
| --- | --- | --- | --- | --- |
| HCT116 | siNC | 1.66 | 0.4189 | 0.9753 |
| HCT116 | siNSUN3 | 0.70 | 0.9870 | 0.9906 |
| DLD1 | siNC | 3.31 | 0.2095 \| | 0.9587 \| |
| DLD1 | siNSUN3 | 1.78 | 0.3902 | 0.9959 |
